# Long-Term Recovery of the Fecal Microbiome and Metabolome of Dogs with Steroid-Responsive Enteropathy

**DOI:** 10.3390/ani11092498

**Published:** 2021-08-25

**Authors:** Rachel Pilla, Blake C Guard, Amanda B Blake, Mark Ackermann, Craig Webb, Steve Hill, Jonathan A Lidbury, Jörg M Steiner, Albert E. Jergens, Jan S Suchodolski

**Affiliations:** 1 Gastrointestinal Laboratory, Department of Small Animal Clinical Sciences, Texas A&M University, College Station, TX 77843, USA; bguard@cvm.tamu.edu (B.C.G.); ABlake@cvm.tamu.edu (A.B.B.); JLidbury@cvm.tamu.edu (J.A.L.); JSteiner@cvm.tamu.edu (J.M.S.); JSuchodolski@cvm.tamu.edu (J.S.S.); 2 Department of Biomedical Sciences and Oregon Veterinary Diagnostic Laboratory, Carlson College of Veterinary Medicine, Oregon State University, Corvallis, OR 97331, USA; Mark.Ackermann@oregonstate.edu; 3 Department of Clinical Sciences, Colorado State University Veterinary Teaching Hospital, Fort Collins, CO 80523, USA; craig.webb@colostate.edu; 4 Veterinary Specialty Hospital by Ethos Veterinary Health, San Diego, CA 92121, USA; shill@ethosvet.com; 5 Flagstaff Veterinary Internal Medicine Consulting, Flagstaff, AZ 86003, USA; 6 Department of Veterinary Clinical Sciences, College of Veterinary Medicine, Iowa State University, Ames, IA 50010, USA; ajergens@iastate.edu

**Keywords:** chronic diarrhea, idiopathic inflammatory bowel disease, microbiota, dysbiosis

## Abstract

**Simple Summary:**

The impact of treatment of dogs with steroid-responsive enteropathy (a form of chronic diarrhea that responds to treatment with a corticosteroid) on the intestinal microbiome (the collection of all microorganisms in the GI tract) and metabolome (the collection of all small molecules produced by the microbiome or the host) was investigated in this study. Dogs receiving standard treatment were evaluated before (week 0), during (week 3), and at the end of treatment (week 8), as well as 1 year later, in comparison to healthy control dogs. None of the dogs had clinically relevant signs of disease at the end of treatment. While both the microbiome and the metabolome normalized to some degree after treatment, some key bacteria and many molecules remained different, suggesting that certain abnormalities persisted. However, 1 year after treatment, both the microbiome and the metabolome were no longer different from healthy dogs, suggesting that intestinal recovery from chronic steroid-responsive enteropathy takes longer than resolution of clinical signs would suggest.

**Abstract:**

The long-term impact of treatment of dogs with steroid-responsive enteropathy (SRE) on the fecal microbiome and metabolome has not been investigated. Therefore, this study aimed to evaluate the fecal microbiome and metabolome of dogs with SRE before, during, and following treatment with standard immunosuppressive therapy and an elimination diet. We retrospectively selected samples from 9 dogs with SRE enrolled in a previous clinical trial, which received treatment for 8 weeks, and had achieved remission as indicated by the post-treatment clinical scores. Long-term (1 year) samples were obtained from a subset (5/9) of dogs. Samples from 13 healthy dogs were included as controls (HC). We evaluated the microbiome using 16S rRNA sequencing and qPCR. To evaluate the recovery of gut function, we measured fecal metabolites using an untargeted approach. While improvement was observed for some bacterial taxa after 8 weeks of treatment, several bacterial taxa remained significantly different from HC. Seventy-five metabolites were altered in dogs with SRE, including increased fecal amino acids and vitamins, suggesting malabsorption as a component of SRE. One year after treatment, however, all bacterial species were evaluated by qPCR and 16S rRNA gene sequencing, and all but thirteen metabolites were no longer different from healthy controls.

## 1. Introduction

Chronic inflammatory enteropathy (CIE) is a common disorder in dogs. CIE in dogs is generally classified according to the response to treatment as food-responsive enteropathy (FRE), antibiotic-responsive enteropathy (ARE), and steroid- or immunosuppressant-responsive enteropathy (SRE, also known as idiopathic inflammatory bowel disease or IBD). Despite different responses to treatment, most dogs with CIE present with intestinal inflammation and alterations of the gut microbiome [1]. Such alterations in composition of the gut microbiota result in functional changes and are referred to as dysbiosis [2]. However, dysbiosis is not limited to patients with SRE, but is also present in other conditions, including acute diarrhea [1,3,4], CIE in general [5,6,7], or exocrine pancreatic insufficiency, and can also be induced by external factors, such as antibiotic administration [8,9,10,11].

An increased abundance of facultative anaerobic bacteria of the family Enterobacteriaceae is often associated with dysbiosis in humans [12], and dogs [5]. In dogs, keystone bacteria are also impacted by dysbiosis, including decreases in the abundance of short-chain fatty acid (SCFA)-producing bacteria such as *Faecalibacterium* spp. and *Bacteroides* [1]. SCFAs are the main energy source for colonocytes, and are decreased in dogs with CIE [13]. In addition to SCFA, fecal metabolomics has revealed other metabolites that are affected by CIE and dysbiosis. Bile acid dysmetabolism has been reported in dogs with CIE [14,15] and with dysbiosis induced by antibiotics [9,10]. Previous studies have shown that bile acid dysmetabolism correlated with decreases in the abundance of *Clostridium hiranonis*, a bacterial species known to convert primary to secondary bile acids [16]. In dogs with FRE, improvement of clinical signs following diet change correlated with increased *C. hiranonis* abundance and normalization of BA levels [17].

Disturbances in other metabolic pathways have been reported in dogs with CIE, including increased fecal lactate [15] and alterations in amino acid metabolism [18,19]. Dogs with SRE [7] and protein-losing enteropathy [18] have been shown to have lower serum tryptophan concentrations. Similar results have been reported for cats [20] and people [21] with CIE, in which tryptophan levels were inversely correlated with the severity of symptoms. When predicted functional composition of the gut microbiome was evaluated, the abundance of genes involved in amino acid metabolism was significantly decreased in dogs with SRE [7].

While dogs with SRE show clinical improvement after treatment, inflammation can still be observed on histological examination [22,23,24]. A previous study [7] in dogs with SRE treated with immunosuppressive therapy found that, despite clinical recovery, microbiome diversity did not improve after 3 weeks of therapy. Long-term changes of the microbiome after treatment, and the impact of treatment on the fecal metabolome have not been investigated. Therefore, the objective of this study was to evaluate the recovery of the fecal microbiome and metabolome of dogs with SRE during and following treatment with standard immunosuppressive therapy and an elimination diet.

## 2. Materials and Methods

Fecal samples from dogs with steroid-responsive enteropathy (SRE) from a previously published clinical trial that evaluated the impact of a probiotic [25] were retrospectively selected. The original clinical trial was approved by the Iowa State University (ISU) Institutional Animal Care and Use Committee (IACUC log # 9–14–7859-K). Because the current study included only archived and spontaneously passed samples, no further approval was required.

Briefly, patients enrolled in the clinical trial had been diagnosed with SRE (idiopathic inflammatory bowel disease, or IBD) [25] by a board-certified veterinary internist based on the World Small Animal Veterinary Association (WSAVA) criteria: chronic GI signs (>3 weeks), histopathologic evidence of mucosal infiltration with inflammatory cells, inability to document other causes of GI inflammation, inadequate response to dietary, antibiotic, and anthelmintic therapies. Exclusion criteria included dogs with other causes for GI signs besides idiopathic SRE, and treatment with antimicrobials, anti-inflammatory drugs, or both within 14 days of presentation. In the clinical trial [25], dogs had been randomized to receive either standard therapy (ST, defined as elimination diet, Purina HA or Royal Canin hydrolyzed protein HP wet and/or dry, fed exclusively for the duration of trial and prednisone PO at a dosage of 0.5–1 mg/kg q12h × 3 weeks then 0.5 mg/kg q12h × 3 weeks then maintained or tapered over the 8 week duration of the study) with placebo, or ST combined with a multi-strain probiotic. Multiple intestinal biopsies were collected by endoscopy at baseline and after 8 weeks of treatment from the stomach, duodenum, ileum, and colon, and histopathology was performed [25].

For the current study, all dogs selected (*n* = 9) had been assigned to receive the standard treatment (hydrolyzed diet + prednisone) which served as placebo in the previous clinical trial, and enough left-over fecal sample was available to complete all the assays required. Each of the dogs had completed the 8-week treatment protocol, achieved remission by the end of the trial, and was completely weaned off prednisone. Samples selected were collected at enrollment (baseline), 3 weeks after the initiation of therapeutic intervention, and 8 weeks after the initiation of therapeutic intervention. Owners were contacted by phone approximately one year later, and a follow-up fecal sample was requested. Owners of 5/9 dogs complied, and therefore, a subset of 5 dogs was evaluated at the long-term follow-up.

Fecal samples from healthy dogs (*n* = 13) belonging to students and staff at the College of Veterinary Medicine & Biomedical Sciences at Texas A&M University were collected. All fecal samples were freely passed, and immediately stored at −80 °C after collection until further analysis. Supplementary Table S1 lists the sex, age, and breed for all dogs included in the study.

### 2.1. Microbiome Analysis

#### 2.1.1. 16S rRNA Gene Sequencing

DNA was extracted from fecal samples using a MoBio Power soil DNA isolation kit (MoBio Laboratories, Carlsbad, CA, USA) following the manufacturer’s instructions [26]. Amplification and sequencing of the V4 variable region 16S rRNA gene was performed at MR DNA (http://www.mrdnalab.com, Shallowater, TX, USA), with forward primer 515F (GTGCCAGCMGCCGCGGTAA) and reverse primer 806R (GGACTACVSGGGTATCTAAT) as previously described [27]. Sequences were processed and analyzed using a Quantitative Insights Into Microbial Ecology 2 (QIIME 2) [28] v 2018.6 pipeline. The raw sequences were uploaded to NCBI Sequence Read Archive under project number SRP122536. Briefly, the sequences were demultiplexed and the amplicon variant sequence (ASV) table was created using DADA2 [29]. Prior to downstream analysis, sequences assigned as chloroplast, mitochondria, and low abundance ASVs, containing less than 0.01% of the total reads in the dataset were removed. All samples were rarefied to even sequencing depth, based on the lowest read depth of samples, to 69,301 sequences per sample.

Alpha (α) diversity was assessed as a measure of richness and evenness within samples, with Chao1, Shannon index, and Observed ASVs data and plots generated with QIIME2. Phylogeny-based unweighted and weighted UniFrac distance metrics were calculated with QIIME2, and used as a measure of beta (β)- diversity to investigate differences between microbial communities.

#### 2.1.2. Dysbiosis Index

To calculate a qPCR-based DI, quantitative PCR assays were performed for total bacteria, *Faecalibacterium*, *Turicibacter*, *Escherichia coli*, *Streptococcus*, *Blautia*, *Fusobacterium*, and *Clostridium hiranonis* as previously described [30,31]. Additionally, a probe-based qPCR assay was performed for *Clostridium perfringens* as previously described [31].

### 2.2. Untargeted Metabolomics

Fecal samples were lyophilized and approximately 10 mg was sent to the West Coast Metabolomics Center (WCMC) at University of California at Davis (http://metabolomics.ucdavis.edu/). Samples were analyzed as a single batch on a gas chromatography time-of-flight mass spectrometry (GC–TOF–MS) platform, in accordance with published methods [32]. Briefly, samples underwent homogenization and extraction, followed by centrifugation. Dried supernatant was resuspended in methanol/chloroform and internal standards were added, followed by drying and derivatization by methoxyamine hydrochloride and N-methyl-N-trimethylsilyltrifluoroacetamide. A volume of 0.5 μL was injected in splitless mode onto a Restek rtx5SilMS column on a temperature-gradient programmed GC (oven 50–330 °C at 20 °C/min, injector 50–250 °C at 12 °C/s) coupled with a Leco Pegasus IV mass spectrometer (scanning 70 spectra/s from 80 to 500 Da, −70 eV ionization energy, 1800 V detector voltage) with helium carrier gas (1 mL/min). Raw data files were processed using ChromaTOF v. 2.32 (Leco, St. Joseph, MI, USA). BinBase algorithm matched spectra to database compounds, and quantification was reported by peak height of an ion at the specific retention index characteristic of the compound across all samples. Peak heights were normalized by average total peak-sums for identified compounds across each sample group. Metabolomics data were uploaded to metabolomicsworkbench.org (submission ST001247).

### 2.3. Statistical Analysis

Clinical disease severity (CIBDAI) scores were analyzed with mixed-effects analysis with the Geisser–Greenhouse correction, followed by Dunnett’s multiple comparison test, in GraphPad Prism 8 (GraphPad Software, San Diego, CA, USA). Histological inflammation scores at baseline and 8 weeks were compared using a paired *t*-test in GraphPad Prism 8.

For 16S rRNA gene sequencing data, univariate statistics were performed using JMP Pro 12 (Cary, NC, USA) for unpaired analysis (Chi Square), and GraphPad Prism 8 was used for paired comparisons (mixed effects analysis with the Geisser–Greenhouse correction, followed by Holm–Sidak’s multiple comparison test). *p*-values were adjusted for multiple comparisons by the Benjamini & Hochberg FDR. Statistical significance was set at q < 0.05. Multivariate statistical analysis for beta diversity (analysis of similarity, ANOSIM) was performed using Primer6 on the UniFrac distance matrixes, both weighted and unweighted.

Fecal DI and qPCR results were analyzed using GraphPad Prism 8 for unpaired comparisons (Kruskal–Wallis test) between SRE and HC groups. *p*-values were adjusted for multiple comparisons by the Benjamini & Hochberg FDR. Statistical significance was set at q < 0.05.

For metabolomics data, univariate and multivariate statistical analysis was carried out using MetaboAnalyst 5.0 [33]. Briefly, the peak height data table was filtered to exclude metabolites of unknown identity and uploaded to MetaboAnalyst 5.0 (Xia Lab, McGill University, Quebec, QC, Canada). The data were log-transformed and pareto scaled before statistical analysis. Principal components analysis (PCA) was performed within MetaboAnalyst. The top 25 features were used to generate a heatmap to visualize metabolomic variability, using Euclidean distance measures and Ward’s algorithm for linkage. Statistics were performed with one-way ANOVA adjusted for multiple comparisons, followed by post-hoc analysis (Tukey’s HSD). Statistical significance was set at *p* < 0.05.

## 3. Results

### 3.1. Clinical Data

Clinical disease severity (CIBDAI) at baseline was scored as moderate-to-severe, with a mean of 8.7 out of a maximum possible score of 18 (SD 3.7). After 3 and 8 weeks of treatment, scores had significantly decreased to 2.2 (SD 4.3, *p* = 0.004) and 0.7 (SD 1.7, *p* < 0.001), respectively. No formal scoring was performed at the long-term follow-up, however, owners of all 9 dogs reported that clinical disease severity remained insignificant, and no further treatment had been performed. None of the 9 dogs were receiving prednisone at this time, and their diets were adjusted at the discretion of the attending clinician. Histopathology findings at baseline and 8 weeks, and inflammation scores based on those findings are shown in Table 1. No significant difference was observed between inflammation scores (*p* = 0.096) before and after treatment.

### 3.2. Microbiome

#### 3.2.1. 16S rRNA Gene Sequencing

The total number of sequences obtained was 4,149,366, with a median of 90,599, minimum of 69,301, and maximum of 127,099. All samples were rarefied to 69,301 sequences for an even depth of analysis.

##### Diversity between Samples (β-Diversity)

**Figure 1:**
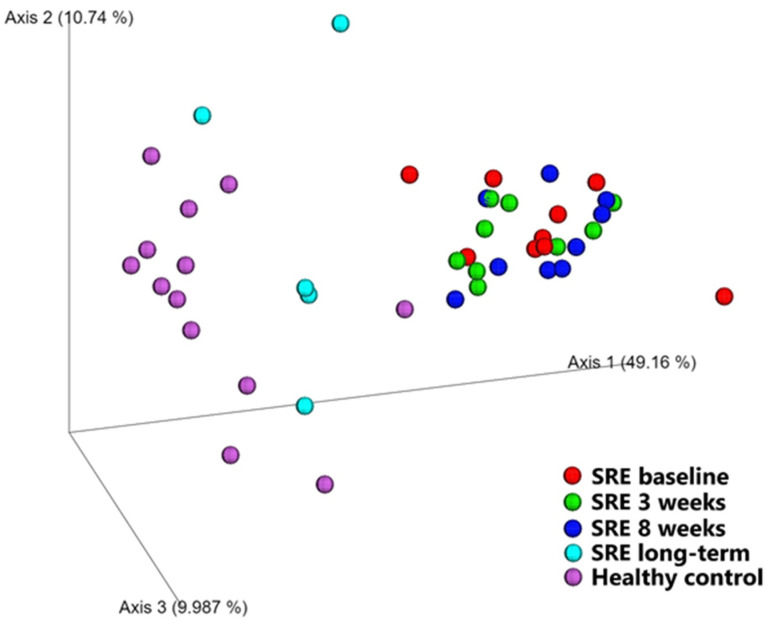
PCoA plot based on weighted UniFrac distances. Samples from healthy control dogs (pink dots) are seen towards the left of the plot, while samples from dogs with SRE at baseline (red dots) can be seen towards the right. While no significant changes from baseline were observed after 3 weeks (green dots, *p* = 0.2) or 8 weeks (blue dots, *p* = 0.2), samples collected 1 year after the initiation of treatment (cyan dots) were significantly different from baseline (*p* < 0.001) and no longer significantly different from healthy control dogs (*p* = 0.1).

Beta diversity, a measurement of diversity between samples, was evaluated qualitatively (unweighted UniFrac) and quantitatively (weighted UniFrac, Figure 1) as shown in Table 2. Both metrics showed that the microbiome of dogs diagnosed with SRE before treatment was significantly different from that of healthy dogs (unweighted UniFrac *p* = 0.002; weighted UniFrac *p* < 0.001). No changes compared to baseline were observed in dogs with SRE after 3 and 8 weeks of treatment, and their microbiome remained significantly different from healthy controls (*p* = 0.001).

For the 1-year follow-up samples, however, no significant difference was found between dogs with SRE and healthy controls, both qualitatively (*p* = 0.12) and quantitatively (*p* = 0.14). Interestingly, when compared with baseline, the microbiome of dogs with SRE one year after initiation of treatment was not changed qualitatively (*p* = 0.76), changing only quantitatively (*p* < 0.001), suggesting changes affected mostly the abundance of the bacterial species present rather than the combination of species present.

##### Diversity within Samples (α-Diversity)

Alpha diversity, i.e., the diversity within samples, was evaluated with Chao1 and Observed ASVs as measurements of richness, and Shannon Index as a measurement of evenness. None of the considered metrics were significantly different from healthy controls at any of the time points (q = 0.67 for all, Figure S1). In addition, none of the follow-up samples from dogs with SRE differed from baseline samples (q = 0.30, q = 0.30, and q = 0.99, for 3 weeks, 8 weeks, and 1-year post initiation of treatment, respectively).

##### Individual Bacterial Taxa

**Figure 2:**
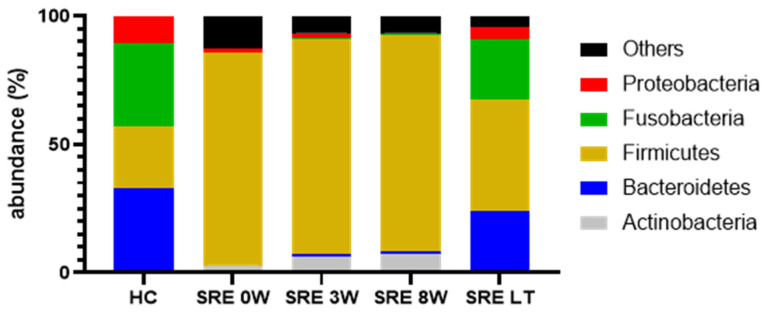
Median abundance of the 5 major bacterial phyla in fecal samples from healthy controls (HC) and dogs with steroid-responsive enteropathy (SRE). While there was no visible change in the microbiome composition of dogs with SRE after up to 8 weeks of treatment, a significant recovery can be seen for the long-term (LT) follow up, and the abundance of all 5 phyla was no longer significantly different from healthy controls.

At baseline, dogs with SRE had significantly different bacterial taxa than the HC group, with significant differences in all 5 main phyla (Figure 2), with increased abundance of Actinobacteria and Firmicutes (q = 0.042 and q < 0.001, respectively) and decreased abundance of Bacteroidetes, Fusobacteria, and Proteobacteria (q < 0.001, q = 0.002, and q = 0.038, respectively), compared to healthy controls. Despite improvement of clinical signs, the response to treatment was not reflected by an improvement of the abundance of any of those phyla, which remained unaltered and significantly different from healthy controls after 8 weeks of treatment. However, after 1 year from the enrollment in the clinical trial, all 5 phyla had recovered at least partially, and were no longer significantly different from healthy controls. A table with median abundance, range, and *p*- and q- values from the most abundant taxa for each taxonomic level is available as Supplementary Material (Table S2).

The increased abundance of Firmicutes was driven mostly by unidentified species within the order Lactobacillales (q = 0.006), which, despite a median reduction from 25% to 5.9% after 8 weeks of treatment, was still significantly different from HC (0.5%, q = 0.003). At the long-term follow up, while the median had only a minor change (5.8%), dogs with SRE were no longer significantly different from HC (q = 0.1). While not significantly different at baseline, abundances of Clostridiaceae and Lachnospiraceae were higher compared to healthy controls. Treatment, however, induced a further increase in these families, and after 3 weeks of treatment both Clostridiaceae (q = 0.025) and Lachnospiraceae (q < 0.001) were significantly higher in the SRE group, and remained so after 8 weeks of treatment (q < 0.001 for both). Both families were no longer significantly different from controls at the long-term follow up (q = 0.44 and q = 0.43). Despite overall increases in abundance of Firmicutes, a few key families had lower abundance in dogs with SRE at baseline, including Ruminococcaceae (q = 0.03) and Veillonellaceae (q = 0.007). Both families responded more quickly to treatment and were no longer significantly different from HC after 3 weeks (q = 0.84 and q = 0.24, respectively).

The decrease of abundance of Bacteroidetes was driven mostly by the families Bacteroidaceae (q = 0.007) and Prevotellaceae (q = 0.003). Both families remained significantly decreased after 8 weeks (q = 0.009 and q < 0.001, respectively), but were no longer significantly different from HC at the long-term follow up (q = 1 and q = 0.42, respectively).

Fusobacteria were severely depleted in dogs with SRE (q = 0.002), with a median abundance of 0.4% at baseline, compared to 32.4% in HC, and were comprised almost exclusively of genus *Fusobacterium* (q = 0.001). As with Fusobacteria, *Fusobacterium* remained significantly decreased after 8 weeks of treatment (q = 0.005) but recovered on the long-term, and was no longer significantly different from HC after 1 year from the beginning of treatment (q = 0.58).

Proteobacteria were significantly decreased in SRE at baseline, driven by genus *Sutterella* (family Alcaligenaceae), whose median abundance at baseline in dogs with SRE was 0.1%, compared to 8% in HC (q = 0.005). *Sutterella* abundance did not improve up to 8 weeks into treatment (0.1%, q = 0.001), but was no longer significantly different from HC in the long-term follow up samples (2.3%, q = 0.32).

#### 3.2.2. qPCR and DI

**Figure 3:**
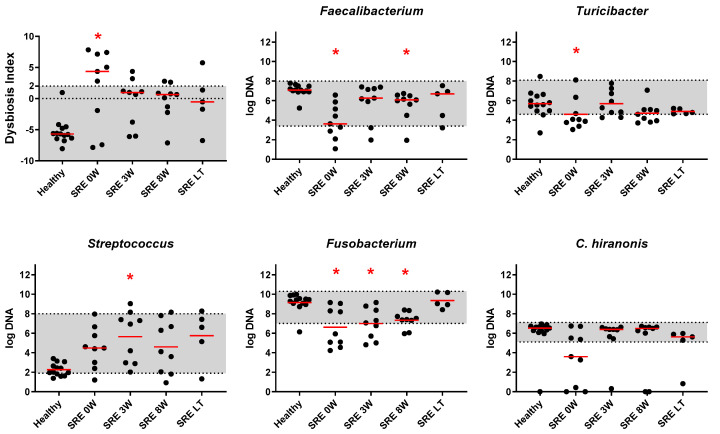
Fecal Dysbiosis Index (DI) and results of qPCR for selected keystone species. Grey shaded areas represent the reference intervals for DI and individual taxa. Red asterisks indicate statistical significance (*p* < 0.05) in comparison to healthy controls, calculated through Kruskal-Wallis test followed by Dunn’s multiple comparisons test. Red lines indicate median.

Prior to treatment, dogs with SRE presented with a significantly increased dysbiosis index (q = 0.007, Figure 3), with 6/9 dogs falling outside of the reference interval (above 2). For interpretation of the DI, values above 2 are considered indicative of dysbiosis, values between 0 and 2 are classified as minor dysbiosis, and values below 0 indicate no detectable dysbiosis. Within the bacterial groups included in the DI, *Faecalibacterium* sp. and *Fusobacterium* spp. were significantly reduced (Figure 3, q < 0.001 and q = 0.002, respectively). *Streptococcus* spp. and *Blautia* spp., instead, were increased but this difference did not reach significance (Figure 3, q = 0.13 and q = 0.52, respectively). The abundance of *Turicibacter* spp. was decreased at baseline (q = 0.043, Figure 3), but was no longer significantly different after 3 weeks of treatment (q > 0.99). The abundance of *C. hiranonis* was decreased in some dogs at baseline, but no significance was found (Figure 3, q = 0.061). *E. coli* and total bacteria were not significantly altered (q > 0.99 for both).

After 3 weeks of treatment with prednisone, the DI decreased and was no longer significantly different from healthy controls (q = 0.068), however, with 2/9 dogs remaining above the reference interval, and 4/9 decreasing to the equivocal range. Despite improvement of DI and CIBDAI scores, only the abundance of *Faecalibacterium* spp. was no longer significantly different from healthy controls (q = 0.4). *Fusobacterium* spp., instead, remained stable and significantly lower than in healthy controls (q = 0.003). *Streptococcus* spp. and *Blautia* spp. showed a further increase, and became significantly higher than healthy controls (q = 0.008 and q = 0.013, respectively).

At the end of 8 weeks of treatment with prednisone, the DI remained stable and not significantly different from healthy controls (q = 0.125), with 2/9 dogs remaining above the reference interval, and 4/9 in the equivocal range. The abundances of *Faecalibacterium* spp. and *Fusobacterium* spp., however, were significantly decreased (q = 0.016 and q = 0.007), and *Blautia* spp. remained significantly increased (q = 0.009), compared to healthy controls. For the 5/9 dogs for which follow-up after 1 year from the beginning of treatment was available, DI (q = 0.26) and all bacterial taxa tested were no longer significantly different from healthy control dogs (Figure 3). Of those, 1/5 dogs remained above the reference interval, and 1/5 dogs was in the equivocal range.

### 3.3. Metabolomics

**Figure 4:**
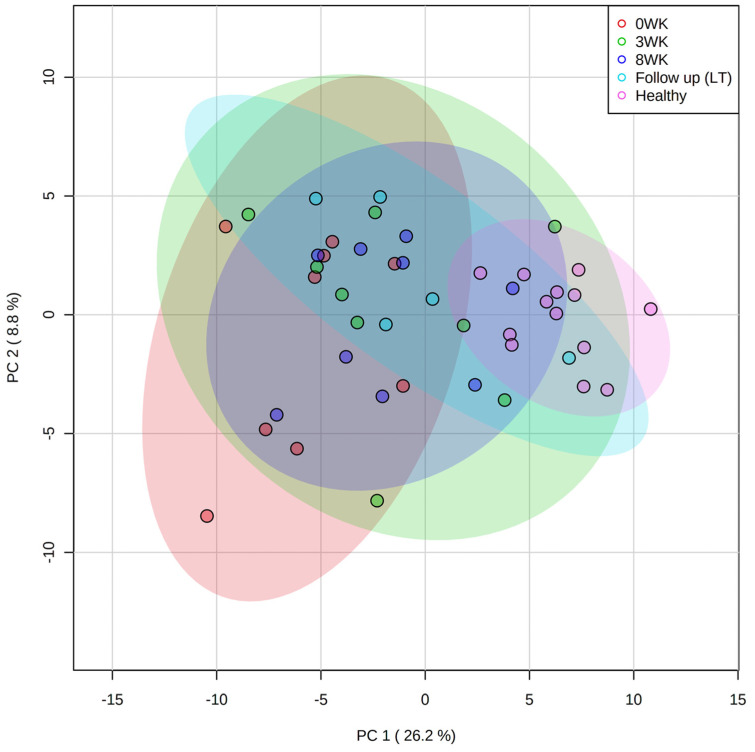
Fecal untargeted metabolomics. PCA plot showing healthy controls (pink dots) being tightly clustered, while samples from dogs with SRE are spread out at baseline (red dots). Shaded ellipses indicate the confidence intervals, with samples becoming progressively more clustered and closer to HC at 3 weeks (green dots), 8 weeks (blue dots), and long-term (LT, 1-year, cyan dots) follow up.

**Figure 5:**
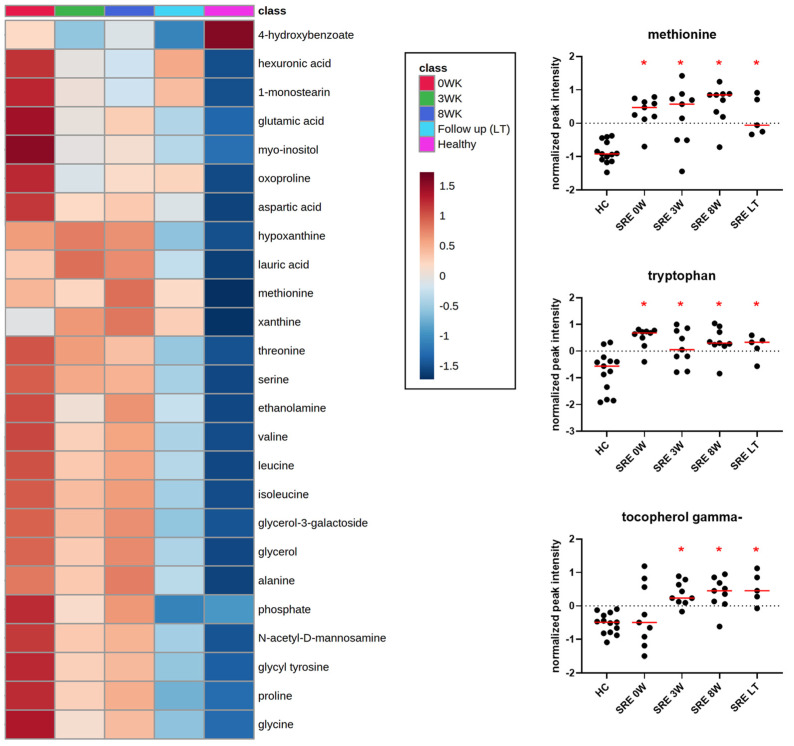
Heatmap of top 25 metabolites (one-way ANOVA adjusted for multiple comparisons) identified in fecal samples (**left**), and relative abundances of methionine, tryptophan, and tocopherol gamma (vitamin E) in HC, and in dogs with SRE over time (**right**). Red asterisks indicate statistical significance (q < 0.05) compared to healthy controls. Note that methionine and tryptophan were significantly increased at baseline and remained increased after 1 year from the beginning of treatment, while tocopherol gamma only increased after the beginning of treatment, and remained increased despite the discontinuation of treatment after 8 weeks.

Untargeted metabolomic analysis identified a total of 664 compounds, 233 of which were named compounds. In the PCoA plot (Figure 4), healthy controls clustered neatly while dogs with SRE before treatment were spread over a wider area. During treatment, the fecal metabolome of the SRE group slowly converged towards the healthy control group (Figure 4), but 1 year after treatment some animals were still separated from the healthy control cluster. A heatmap with the group averages for the top 25 metabolites is shown in Figure 5.

After correction for multiple comparisons, 75 compounds were found to be significantly different from controls at baseline (Table 3, Table S3, and Figure S2). Sixteen amino acids and 3 dipeptides were significantly increased in dogs with SRE at baseline, including proline, isoleucine, leucine, alanine, and tryptophan. Thirteen amino acids remained significantly increased after 8 weeks of treatment. However, at 1-year follow up, all amino acids except methionine and tryptophan (Figure 5) were no longer significantly different from controls. Other increased metabolites included glucose and glucose metabolites (e.g., gluconic acid and ribose); monosaccharides (e.g., fructose and xylulose); vitamin C metabolites (threonic and isothreonic acid); and fatty acids (e.g., stearic acid, caprylic acid, and arachidonic acid). Thirteen metabolites were significantly different from controls after 1 year, 3 of which (maltose, indole-3-lactate, and tocopherol gamma) were not significant at baseline but became significantly altered during treatment.

## 4. Discussion

This study aimed to evaluate the recovery of the fecal microbiome and metabolome of dogs with SRE over time, during and following treatment with standard immunosuppressive therapy and an elimination diet. We retrospectively selected samples from 9 dogs with SRE that had been enrolled in a previous clinical trial [25]. These dogs had received treatment with prednisone and an elimination diet for a period of 8 weeks, and had achieved remission as indicated by the post-treatment clinical scores. Long-term (1-year after initiation of treatment) samples were obtained from a subset (5/9) of dogs. We evaluated the microbiome using 16S rRNA sequencing and qPCR. To evaluate the recovery of gut function, we measured fecal metabolites using an untargeted metabolomics approach. While improvement was observed for some bacterial taxa and the fecal DI after 8 weeks of treatment, several bacterial taxa and metabolites remained significantly different from HC. However, 1 year after initiation of treatment, the abundance of all bacterial species measured by qPCR and 16S rRNA gene sequencing, and all but thirteen metabolites were no longer significantly different from healthy controls.

Dysbiosis is associated with increased levels of oxygen in the intestinal lumen [34], which could be due to increased gut permeability and/or gut inflammation [12]. The increase in free oxygen negatively impacts strict anaerobe bacterial populations, and leads to a blooming of facultative anaerobe bacteria, including members of the *Enterobacteriaceae* family [2], which can in turn exacerbate inflammation. While the association between dysbiosis and SRE has been extensively described [1,5,6,13,14,15,30,35] and reviewed elsewhere [36], it remains unclear whether the dysbiosis is a symptom of the disease, or a causative factor.

In our study, we observed that dogs with SRE showed a significant increase in the fecal DI (Figure 3), with 6/9 dogs presenting DI values above the reference interval (DI ≥ 2, dysbiosis). While not all keystone bacteria included in the DI recovered during the course of treatment, the combined index (DI) recovered quickly and was no longer significantly different from controls after 3 weeks, with only 2/9 dogs having a DI above the reference interval. After 8 weeks, the abundance of *Faecalibacterium* spp., *Fusobacterium* spp., and *Blautia* spp. were still significantly altered, but at the long-term follow up all 7 keystone bacteria and the fecal DI were no longer significantly different from HC, and only 1/5 dogs had a DI value above the reference interval.

In addition to the fecal DI, we also analyzed the bacterial composition of the fecal microbiome through 16S rRNA gene sequencing. We observed, in agreement with the literature [1,5,7], that dogs with SRE have significant changes in overall microbiome composition at the time of diagnosis, and cluster visibly apart from healthy controls (Figure 1). Similarly to our previous study [7], no improvement was observed after 3 weeks of treatment; furthermore, at the end of treatment (8 weeks) fecal samples still clustered together with baseline samples, despite significant clinical improvement. However, as seen in Figure 1, after 1 year from the initiation of treatment, samples obtained from a subset of dogs clustered with those of healthy control dogs and were no longer statistically significantly different from them. Our results indicate that the recovery of the gut microbiome composition in SRE-associated dysbiosis is a long-term process. In agreement, no difference in histological inflammation scores was observed after 8 weeks of treatment compared to baseline, and 7/9 dogs still presented with some degree of intestinal inflammation. While no biopsies were obtained at the long-term follow up, it is possible that the improvement in overall microbiome composition reflect an improvement in luminal conditions due to decreased intestinal inflammation [2,12], underscoring that the recovery from SRE is a slow process.

Dogs with CIE are also known to present with significantly decreased fecal bacterial diversity, as indicated by the decrease in total bacteria by qPCR [13,30,35,37] and by the decrease in richness and evenness diversity indicators [1,7]. In our study, we did not observe a decrease in total bacteria by qPCR, nor decreases in bacterial diversity in fecal samples from dogs with SRE in any of the time points investigated. It is possible that our study lacked statistical power to identify such differences due to the small number of dogs included.

When individual bacterial taxa were considered, all 5 major phyla were significantly affected by disease. In feces from dogs with SRE, there was a significant increase of Firmicutes and Actinobacteria, and a decrease of Proteobacteria, Fusobacteria, and Bacteroidetes, which were barely detectable at baseline (Figure 2). The increased Firmicutes to Bacteroidetes ratio observed in our study is a common finding in dysbiosis across species and has been described in GI- [38,39] and non-GI-related [40,41] diseases.

In our study, increases in Firmicutes were driven mostly by unidentified species within the order Lactobacillales. Increased lactic acid bacteria abundance has been previously reported in dogs with CIE [13,15]. Lactobacillales decreased with treatment, and while it became not significantly different from HC after 1 year, it remained 10 times higher than HC (5.8% vs. 0.5%). While we did not measure fecal lactate concentrations, dogs with CIE have been reported to have higher fecal lactate concentrations [15], and the role of lactic acid bacteria and fecal lactate in dogs with CIE remains unclear.

Short-chain fatty acid (SCFA) production is an intestinal function known to be depleted in dogs with CIE [13]. Bacteroidetes, a phylum found to be decreased not only at baseline, but also at 3 and 8 weeks from the beginning of treatment, contains genera, such as *Bacteroides* spp. and *Prevotella* spp., that are SCFA-producers. Genus *Bacteroides* reached abundances comparable to the healthy controls after 1 year from treatment. In addition, qPCR results show that *Faecalibacterium*, another SCFA-producing bacterium was also decreased in dogs with SRE at baseline and 8 weeks, but no longer different from HC after 1 year. When considering the reference intervals (RI) for *Faecalibacterium*, however, we can see a quicker recovery of its abundance, going from 4/9 dogs below the RI at baseline to 2/9 at 3 weeks, and 1/9 at 8 weeks, suggesting that treatment was successful in restoring *Faecalibacterium* abundances in most dogs. While the untargeted metabolomics method performed in this study was unsuitable to identify SCFAs, it is likely that SCFAs were at least partially restored by treatment and at the long-term follow up.

Fusobacteria is a major phylum in the fecal microbiome of healthy dogs [36], whose abundance was severely decreased in dogs with SRE (median 0.4% vs. 32.4% in HC, Figure 2), which is in agreement with the literature [1,13,30]. Some *Fusobacterium* species can produce butyrate, a SCFA, from protein, which could explain its role in the healthy microbiome of dogs. *Fusobacterium* remained significantly decreased after 8 weeks of treatment (median 0.6%); however, at the long-term follow-up, the median abundance of *Fusobacterium* was 20.3% and was no longer significantly different from HC.

Proteobacteria, and in particular γ-Proteobacteria, are typically increased in dogs with CIE. Gamma-Proteobacteria are mainly composed of Enterobacteriaceae (e.g., *E. coli*), and their increase has been associated with a number of diseases and dysbiosis. In contrast, in our study we found a significant decrease in Proteobacteria, driven by a depletion of β-Proteobacteria from genus *Sutterella*, in agreement with previous findings in duodenum samples of dogs with SRE [6]. Only a minor and not statistically significant increase in Enterobacteriaceae was observed in our data. Similarly, no changes in *E. coli* abundance were observed in qPCR.

Metabolomics revealed that many metabolic functions are impaired in dogs with SRE, including some previously identified pathways. Sixteen amino acids were found to be increased in the feces of dogs with SRE, a finding that suggests that amino acid absorption may be compromised in these animals. While some amino acids normalized quickly with treatment, 13 amino acids were still increased after 8 weeks of immunosuppressive treatment. Similar results were reported in humans with Crohn’s disease [42], where despite immunosuppressive treatment, several amino acids, including isoleucine, leucine, and alanine were increased in fecal samples. Malabsorption is a known consequence of intestinal inflammation in CIE and is due to the destruction of the brush border [43,44]. Amino acid malabsorption, especially for essential amino acids, however, could compromise healing of the intestinal mucosa, and therefore, approaches to compensate amino acid malabsorption in CIE should be investigated.

Tryptophan is an essential amino acid that plays a significant role in GI health, and is a precursor for compounds such as kynurenine, serotonin, melatonin, and indole [45]. In our study, tryptophan (Figure 5) was significantly increased in feces before, during treatment, and after 1 year. In addition to malabsorption, other mechanisms can affect tryptophan abundance in feces and serum of dogs with CIE. Humans with IBD have been found to have an increased expression of the enzyme indoleamine 2,3, dioxygenase 1 (IDO-1) [46], leading to lower serum tryptophan concentrations due to increased kynurenine production [21]. Increased tryptophan catabolism also limits the production of serotonin, a neurotransmitter that is essential for GI secretion, motility, and pain perception [47], which may be another mechanism through which tryptophan affects GI health. In dogs with protein-losing enteropathy, a severe enteropathy leading to hypoproteinemia, tryptophan was the only serum amino acid found to be decreased, and correlated with serum albumin concentrations [18]. In cats with CIE, serum tryptophan levels were decreased, and inversely correlated with disease severity [20]. Tryptophan and its bacteria-produced indole metabolites have anti-inflammatory properties [48], and in animal models, supplementation with tryptophan ameliorates DSS-induced colitis [49].

Two other essential amino acids of concern that were found to be increased in the feces of dogs with SRE in our study, before and during treatment, were threonine and methionine. Threonine is essential for mucin synthesis and has a role in the maintenance of the intestinal barrier function [50]. Methionine is crucial for the maintenance of intestinal integrity and for intestinal antioxidant capacity [51]. Methionine remained increased at the long-term follow-up, suggesting that its absorption may have remained impaired despite improvement in clinical parameters and gut microbiome composition.

Proline concentrations were also increased in fecal samples from dogs with SRE, not only at baseline but also at 3 and 8 weeks into treatment. While dogs are capable of synthesizing proline, the per-gram requirements of proline for protein synthesis is the highest among all amino acids, and synthesis may not be enough to meet requirements in non-physiological situations [52]. Proline is necessary for tissue repair, having a role in regulating proliferation of intestinal epithelial cells and in expression of tight junctions [53,54]. Therefore, decreased absorption of proline can slow the recovery of the intestinal epithelium, limiting the efficacy of treatment.

Vitamin pathways were also affected. Nicotinic acid (vitamin B_3_) was increased, albeit not significantly, in fecal samples of dogs with SRE. Interestingly, however, the levels of nicotinic acid increased further, and were significantly increased after 3 and 8 weeks of treatment, returning to values similar to HC before the 1 year follow up. Nicotinic acid can be produced by the host and by bacteria from tryptophan [55], and its increase could be a consequence of the increased availability of luminal tryptophan. A previous study found nicotinic acid to be increased also in the serum of dogs with SRE [7]. Similarly, vitamin E (tocopherol delta and gamma) was also increased at weeks 3 and 8, and tocopherol gamma still remained increased after 1 year (Figure 5). Threonic acid, a metabolite from the breakdown of vitamin C, was also increased in dogs with SRE. While threonic acid levels in feces were significantly decreased with treatment, at the 1 year follow up threonic acid was again found to be significantly increased, driven by 2/5 dogs with levels as high or higher than their baseline values. The role of threonic acid in feces is unknown; however, its increase could be indicative of increased vitamin C degradation due to increased oxidative stress.

This study has limitations that need to be considered. The main limitation was the small number of animals included, which was a direct result of the strict criteria for inclusion. Enrollment of well-defined SRE cases is notoriously difficult, and in addition, we retrospectively included only dogs (*n* = 9) that presented a significant improvement with standard therapy and whose clinical scores were considered insignificant after 8 weeks of therapy. For the 1-year follow-up, only 5/9 owners replied to a request for follow-up samples, which further reduced our statistical power. In addition, while the microbiome of healthy controls is stable over time in the absence of interventions, and therefore, a single sample from controls is adequate for the comparisons made in the study [56], the variability of the fecal metabolome of healthy dogs over time is unknown. However, a large proportion of the metabolites found in fecal samples are microbiome-derived or microbiome-modulated, and important pathways and metabolic functions must be conserved to preserve physiological functioning of the GI tract. Future studies investigating the variation of the fecal metabolome of healthy dogs over time should be conducted.

## 5. Conclusions

We were able to describe how the recovery of the gut microbiome did not coincide with clinical remission in a small cohort of dogs with SRE. Increases in abundance of amino acids and vitamins in fecal samples of dogs with SRE suggest the occurrence of malabsorption that is not always quickly and fully restored with treatment, and it is possible that this may limit further intestinal healing. However, we demonstrated that the recovery of the gut microbiome and metabolome, both in composition and functionality, is an achievable, albeit long-term goal. A healthy and functional microbiome is believed to be essential for the maintenance of health, and is potentially a goal to prevent clinical relapses. Further studies of gastrointestinal diseases that include a microbiome component should strive to obtain longer follow-up periods that encompass not only clinical improvement but also microbiome recovery to evaluate is correlation with long-term remission.

## Figures and Tables

**Table 1:** Histopathology findings and histological inflammation scores at baseline and after 8 weeks of treatment. Biopsies were collected from the stomach, duodenum, ileum and colon. Scores range from 0 to 3, where 0 = no inflammation, 1 = mild inflammation, 2 = moderate inflammation, and 3 = severe inflammation. No significant difference was found between baseline and 8 weeks (paired *t*-test, *p* = 0.096). Mild-mod = mild-to-moderate; L = lymphocytic; P = plasmacytic; LP = lymphocytic-plasmacytic; IELs = intra-epithelial lymphocytes.

Patient and Time Point	Histopathologic Diagnosis	Cellular Infiltrates	Mucosal Architecture	Inflammation Scores
**Dog 1** Baseline	Mild-mod LP gastritis; mild-mod LP enteritis	Lymphocytes, plasma cells	No abnormalities	3
8 weeks	Mild-mod LP gastritis; mod-severe LP enteritis	Lymphocytes, plasma cells, eosinophils	Multi-focal lacteal dilation	1
**Dog 2** Baseline	Moderate L gastritis; moderate LP enteritis; mild L colitis	Lymphocytes, plasma cells	Lacteal dilation; crypt hyperplasia	2
8 weeks	Mild L gastritis; mild LP enteritis; mild LP colitis	Lymphocytes, plasma cells	Lacteal dilation; crypt hyperplasia	1
**Dog 3** Baseline	Moderate LP enteritis; mild L colitis	Lymphocytes, plasma cells	Lacteal dilation; multi-focal crypt abscesses	1
8 weeks	Mild-mod LP enteritis; mild LP colitis; intestinal lymphangiectasia	Lymphocytes, plasma cells	Lacteal dilation	1
**Dog 4** Baseline	Severe L gastritis with fibrosis; severe L enteritis; moderate L colitis	Lymphocytes	Gastric fibrosis; multi-focal dilated lacteals; increased IELs; crypt hyperplasia; diffuse villus blunting; increased goblet cells	1
8 weeks	Severe LP gastritis; mild-mod LP enteritis; mild LP colitis	Lymphocytes, plasma cells, neutrophils	Tortuous colonic glands	0
**Dog 5** Baseline	Mild LP gastritis; mild-mod LP enteritis	Lymphocytes, plasma cells	Multi-focal dilated lacteals	1
8 weeks	Mild LP enteritis; mild LP colitis	Lymphocytes, plasma cells	No abnormalities	2
**Dog 6** Baseline	Moderate LP enteritis	Lymphocytes, plasma cells	No abnormalities	1 0
8 weeks	Mild LP gastritis; moderate LP enteritis; moderate LP colitis	Lymphocytes, plasma cells	No abnormalities	
**Dog 7** Baseline	Mild LP gastritis; mod plasmactyic enteritis; mild LP colitis	Lymphocytes, plasma cells	Gastric edema; multi-focal crypt abscesses; hyperplasia of colonic glandular epithelium	2
8 weeks	Mild LP gastritis; mild-mod LP enteritis; mild LP colitis	Lymphocytes, plasma cells	Multifocal lacteal dilation; multi-focal crypt abscesses	1
**Dog 8** Baseline	Mild-mod LP enteritis	Lymphocytes, plasma cells	No abnormalities	1
8 weeks	Moderate P enteritis; moderate P colitis with fibrosis	Plasma cells	Multi-focal lacteal dilation; mild colonic fibrosis	1
**Dog 9** Baseline	Moderate LP gastritis; moderate LP enteritis	Lymphocytes, plasma cells	Multifocal lacteal dilation; multifocal crypt abscesses	2
8 weeks	Moderate LP gastritis; moderate LP enteritis	Lymphocytes, plasma cells	No abnormalities	2

**Table 2:** Analysis of similarities (ANOSIM) of beta diversity, performed on qualitative (Unweighted UniFrac) and quantitative (Weighted UniFrac) microbiome composition data. R-values indicate the similarity between groups, with values close to 0 indicating similar microbiome composition, and values close to 1 indicating highly different microbiome composition. LT = long-term follow-up.

Pairwise Comparisons	Unweighted UniFrac		Weighted UniFrac	
	R-Value	*p*-Value	R-Value	*p*-Value
**SRE baseline vs. week 3**	0.014	0.373	0.042	0.198
**SRE baseline vs. week 8**	0.052	0.172	0.063	0.163
**SRE baseline vs. LT**	−0.101	0.761	0.449	0.001
**Healthy vs. SRE baseline**	0.348	0.002	0.780	0.001
**Healthy vs. SRE week 3**	0.410	0.001	0.833	0.001
**Healthy vs. SRE week 8**	0.457	0.001	0.886	0.001
**Healthy vs. SRE LT**	0.168	0.119	0.169	0.141

**Table 3:** Selected fecal metabolites identified by untargeted metabolomics that were significantly different in dogs with SRE (complete list available in Table S3). Compound type, direction of change compared to healthy controls (HC), as well as the time points at which changes were statistically significant are indicated, as well as overall *p*-values and adjusted *p*-values (FDR). NIST = National Institute of Standards and Technology.

Compound Name	Compound Type	Change in Relation to HC	Time Points Significantly Different from HC	*p*-Value	FDR
**alanine**	amino acid	increased	baseline, 3 weeks, 8 weeks	0.000	0.000
**asparagine**	amino acid	increased	baseline	0.001	0.006
**aspartic acid**	amino acid	increased	baseline, 3 weeks, 8 weeks	0.000	0.000
**cysteine**	amino acid	increased	baseline	0.009	0.029
**cystine**	amino acid	increased	baseline	0.001	0.003
**glutamic acid**	amino acid	increased	baseline, 3 weeks, 8 weeks	0.000	0.000
**glycine**	amino acid	increased	baseline, 3 weeks, 8 weeks	0.000	0.000
**isoleucine**	amino acid	increased	baseline, 3 weeks, 8 weeks	0.000	0.000
**leucine**	amino acid	increased	baseline, 3 weeks, 8 weeks	0.000	0.000
**lysine**	amino acid	increased	3 weeks, 8 weeks	0.012	0.037
**methionine**	amino acid	increased	baseline, 3 weeks, 8 weeks, long term	0.000	0.000
**phenylalanine**	amino acid	increased	baseline, 3 weeks, 8 weeks	0.000	0.002
**proline**	amino acid	increased	baseline, 3 weeks, 8 weeks	0.000	0.000
**serine**	amino acid	increased	baseline, 3 weeks, 8 weeks	0.000	0.000
**threonine**	amino acid	increased	baseline, 3 weeks, 8 weeks	0.000	0.000
**tryptophan**	amino acid	increased	baseline, 3 weeks, 8 weeks, long term	0.000	0.001
**valine**	amino acid	increased	baseline, 3 weeks, 8 weeks	0.000	0.000
**glycyl tyrosine**	dipeptide	increased	baseline, 3 weeks, 8 weeks	0.000	0.000
**glycyl-proline**	dipeptide	increased	baseline	0.012	0.037
**homocystine**	dipeptide	increased	baseline	0.018	0.048
**glucose**	glucose	increased	baseline, 3 weeks, 8 weeks	0.000	0.002
**6-deoxyglucose**	glucose metabolite	increased	baseline, 3 weeks, 8 weeks	0.000	0.002
**gluconic acid**	glucose metabolite	increased	baseline	0.012	0.036
**myo-inositol**	glucose metabolite	increased	baseline	0.000	0.001
**ribose**	glucose metabolite	increased	baseline, 3 weeks, 8 weeks	0.000	0.002
**indole-3-lactate**	indole	decreased	3 weeks, 8 weeks, long term	0.000	0.002
**isothreonic acid**	metabolite vitamin C	increased	baseline	0.005	0.019
**threonic acid**	metabolite vitamin C	increased	baseline, long term	0.003	0.014
**fructose**	monosaccharide	increased	baseline, 8 weeks	0.000	0.003
**N-acetyl-D- mannosamine**	monosaccharide	increased	baseline, 3 weeks, 8 weeks	0.000	0.000
**tagatose**	monosaccharide	increased	baseline	0.006	0.022
**xylulose NIST**	monosaccharide	increased	baseline, 3 weeks, 8 weeks	0.003	0.011
**arachidonic acid**	omega 6 fatty acid	increased	baseline, 3 weeks, 8 weeks	0.000	0.002
**arachidic acid**	saturated fatty acid	increased	baseline, 3 weeks	0.002	0.009
**caprylic acid**	saturated fatty acid	increased	baseline, 3 weeks	0.001	0.006
**isoheptadecanoic acid NIST**	saturated fatty acid	increased	baseline, 8 weeks	0.006	0.022
**lauric acid**	saturated fatty acid	increased	baseline, 3 weeks, 8 weeks	0.000	0.001
**lignoceric acid**	saturated fatty acid	increased	baseline	0.001	0.005
**myristic acid**	saturated fatty acid	increased	3 weeks	0.019	0.050
**palmitic acid**	saturated fatty acid	increased	baseline	0.018	0.048
**stearic acid**	saturated fatty acid	increased	baseline, 3 weeks	0.000	0.001
**nicotinic acid**	vitamin B3	increased	3 weeks, 8 weeks	0.002	0.008
**tocopherol delta- NIST**	vitamin E	increased	3 weeks, 8 weeks	0.012	0.036
**tocopherol gamma-**	vitamin E	increased	3 weeks, 8 weeks, long term	0.000	0.002

## Data Availability

The raw sequences obtained from 16S rRNA gene sequencing were uploaded to NCBI Sequence Read Archive under project number SRP122536. Metabolomics raw data was uploaded to metabolomicsworkbench.org (submission ST001247).

## Supplementary Materials

The following are available online at https://www.mdpi.com/article/10.3390/ani11092498/s1, Figure S1: Alpha diversity parameters Chao1, Observed ASVs, and Shannon Index in healthy controls (HC) and dogs with SRE over time. No statistical significance was found. Figure S2: Abundance of fecal metabolites (n = 30) found to be significantly affected by SRE, organized in alphabetic order. Red asterisks indicate statistical significance compared to healthy controls. Table S1: Signalment of all dogs included in the study. Table S2: List of all bacterial taxa identified in fecal samples. Median and range by group and time point, as well as statistical comparisons with healthy controls, and over time. Table S2: Fecal metabolites identified by untargeted metabolomics that were significantly altered by SRE. Compound type, direction of change compared to healthy controls (HC), as well as the time points in which changes were statistically significant are indicated, as well as overall *p*-values and adjusted *p*-values (FDR).
