# Supplementary material for: Long-Term Recovery of the Fecal Microbiome and Metabolome of Dogs with Steroid-Responsive Enteropathy
Source: Animals (Basel). 2021 Aug 25;11(9):2498. doi: 10.3390/ani11092498 (PMC8468387; doi:10.3390/ani11092498)

Supplementary figure S1: Alpha diversity parameters Chao1, Observed ASVs, and Shannon Index in healthy controls (HC) and dogs with SRE over time. No statistical significance was found.

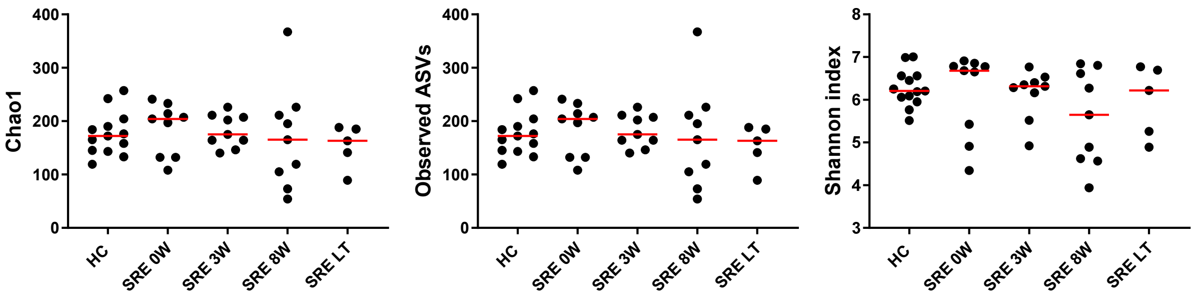

Supplement: Supplementary file 1 [file animals-11-02498-s001.zip › Supplementary_figure_S1_alpha_div.pdf]
