# Supplementary material for: Long-Term Recovery of the Fecal Microbiome and Metabolome of Dogs with Steroid-Responsive Enteropathy
Source: Animals (Basel). 2021 Aug 25;11(9):2498. doi: 10.3390/ani11092498 (PMC8468387; doi:10.3390/ani11092498)
Supplement: Supplementary file 1 [file animals-11-02498-s001.zip › Supplementary_figure_S2_metabolites.pdf]

Supplementary figure S2: Abundance of fecal metabolites (n=30) found to be significantly affected by SRE, organized in alphabetic order. Red asterisks indicate statistical significance compared to healthy controls.

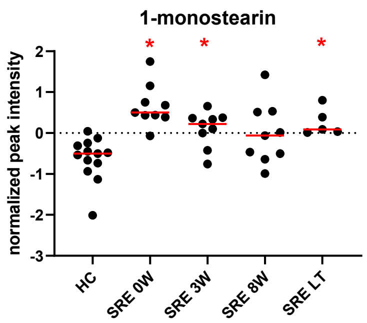
1-monostearin
normalized peak intensity
2
1
0
-1
-2
-3
*
*
*
HC
SRE 0W
SRE 3W
SRE 8W
SRE LT

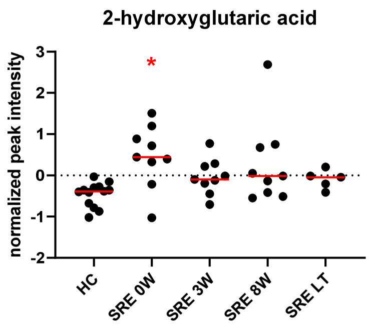
2-hydroxyglutaric acid
normalized peak intensity
3
2
1
0
-1
-2
*
HC
SRE 0W
SRE 3W
SRE 8W
SRE LT

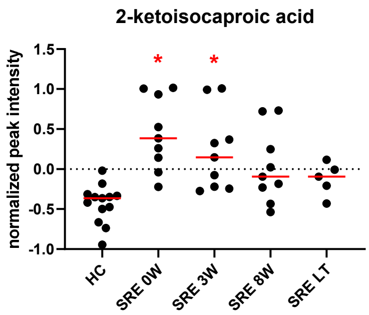
2-ketoisocaproic acid
normalized peak intensity
1.5
1.0
0.5
0.0
-0.5
-1.0
*
*
HC
SRE 0W
SRE 3W
SRE 8W
SRE LT

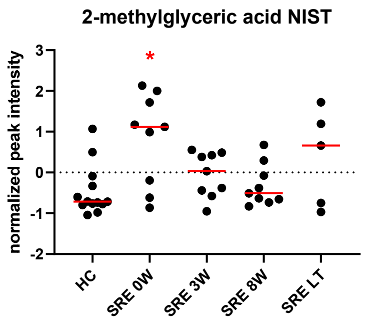
2-methylglyceric acid NIST
normalized peak intensity
3
2
1
0
-1
-2
*
HC
SRE 0W
SRE 3W
SRE 8W
SRE LT

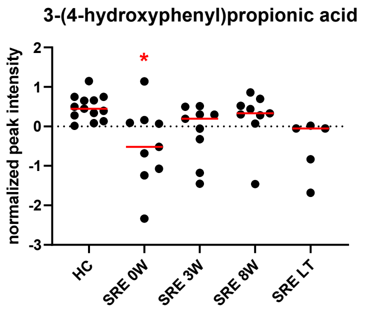
3-(4-hydroxyphenyl)propionic acid
normalized peak intensity
2
1
0
-1
-2
-3
*
HC
SRE 0W
SRE 3W
SRE 8W
SRE LT

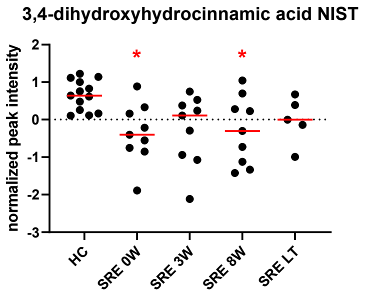
3,4-dihydroxyhydrocinnamic acid NIST
normalized peak intensity
2
1
0
-1
-2
-3
*
*
HC
SRE 0W
SRE 3W
SRE 8W
SRE LT

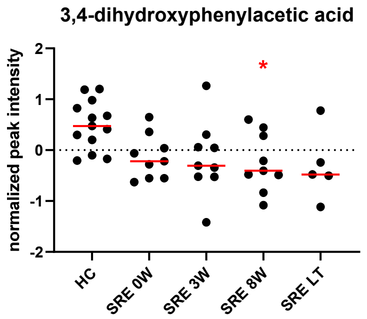
3,4-dihydroxyphenylacetic acid
normalized peak intensity
2
1
0
-1
-2
*
HC
SRE 0W
SRE 3W
SRE 8W
SRE LT

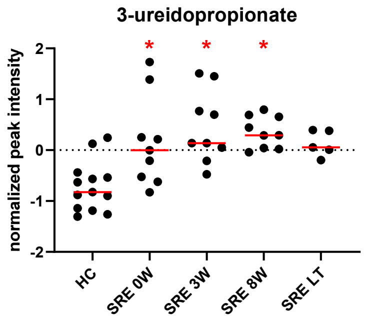
3-ureidopropionate
normalized peak intensity
2
1
0
-1
-2
*
*
*
HC
SRE 0W
SRE 3W
SRE 8W
SRE LT

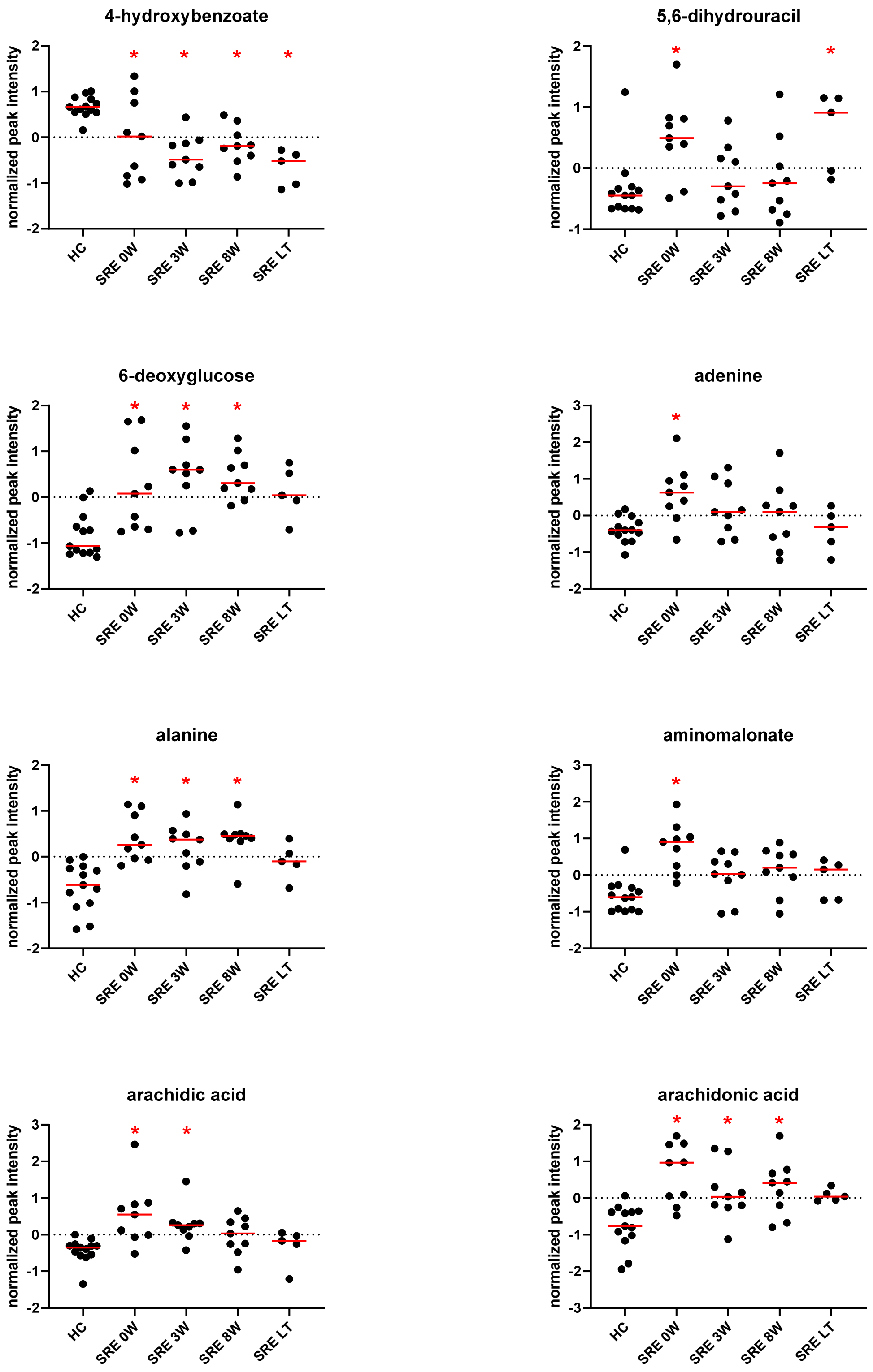
4-hydroxybenzoate
5,6-dihydrouracil
6-deoxyglucose
adenine
alanine
aminomalonate
arachidic acid
arachidonic acid
normalized peak intensity
HC
SRE 0W
SRE 3W
SRE 8W
SRE LT

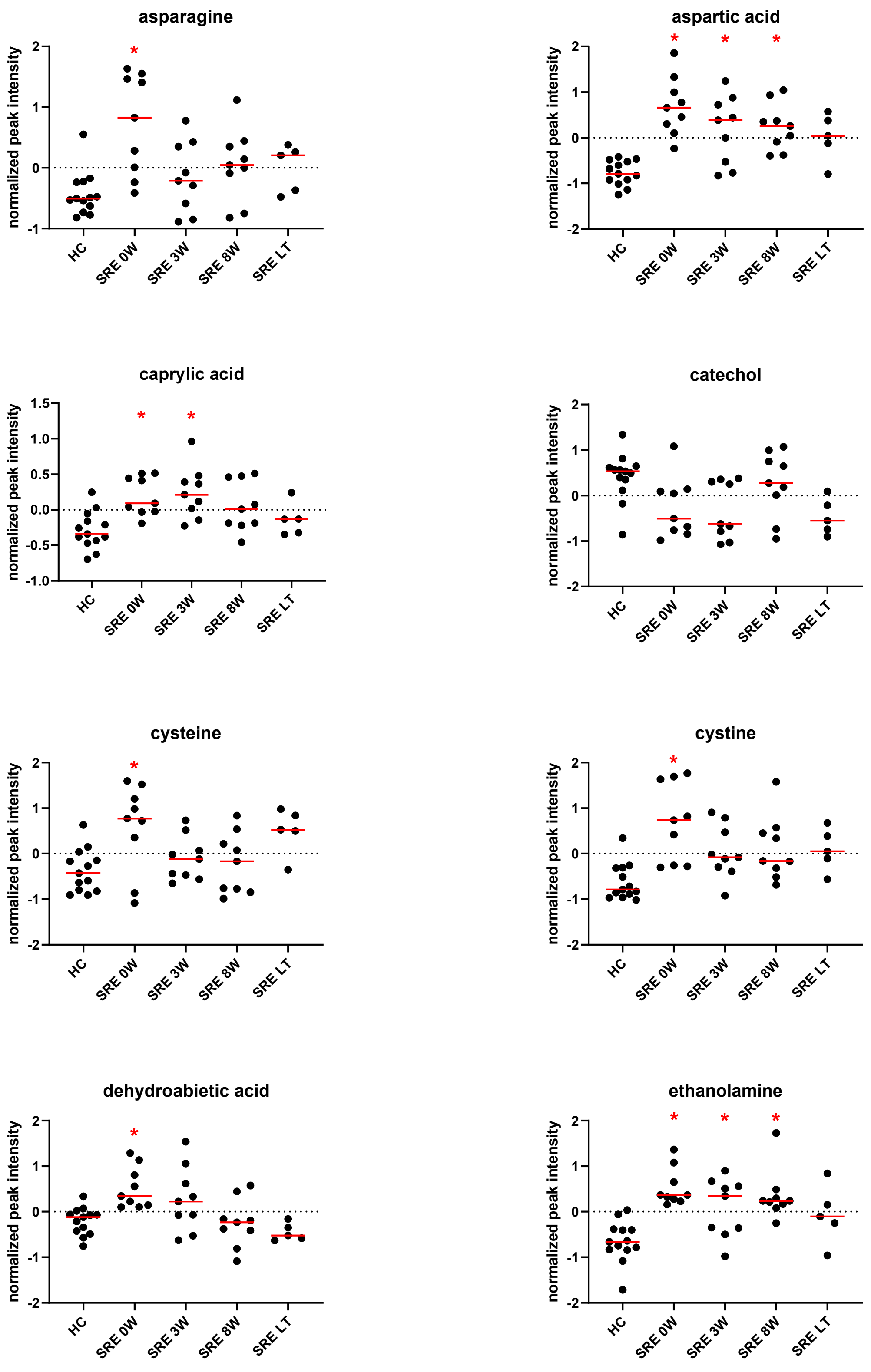
asparagine
aspartic acid
caprylic acid
catechol
cysteine
cystine
dehydroabietic acid
ethanolamine
normalized peak intensity
2
1
0
-1
-2
1.5
1.0
0.5
0.0
-0.5
-1.0
*
HC
SRE 0W
SRE 3W
SRE 8W
SRE LT

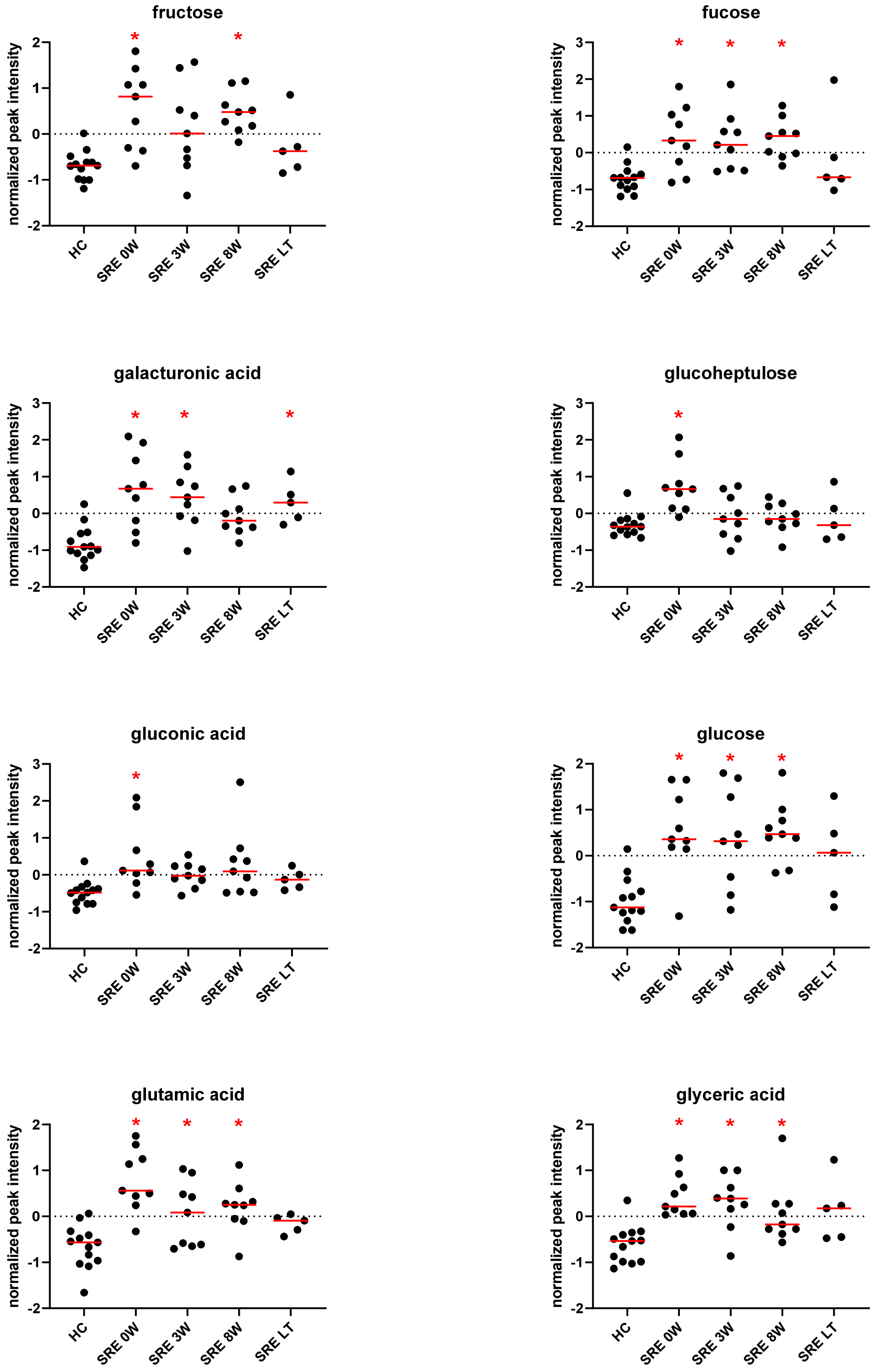
fructose
fucose
galacturonic acid
glucoheptulose
gluconic acid
glucose
glutamic acid
glyceric acid
normalized peak intensity
HC
SRE 0W
SRE 3W
SRE 8W
SRE LT
*

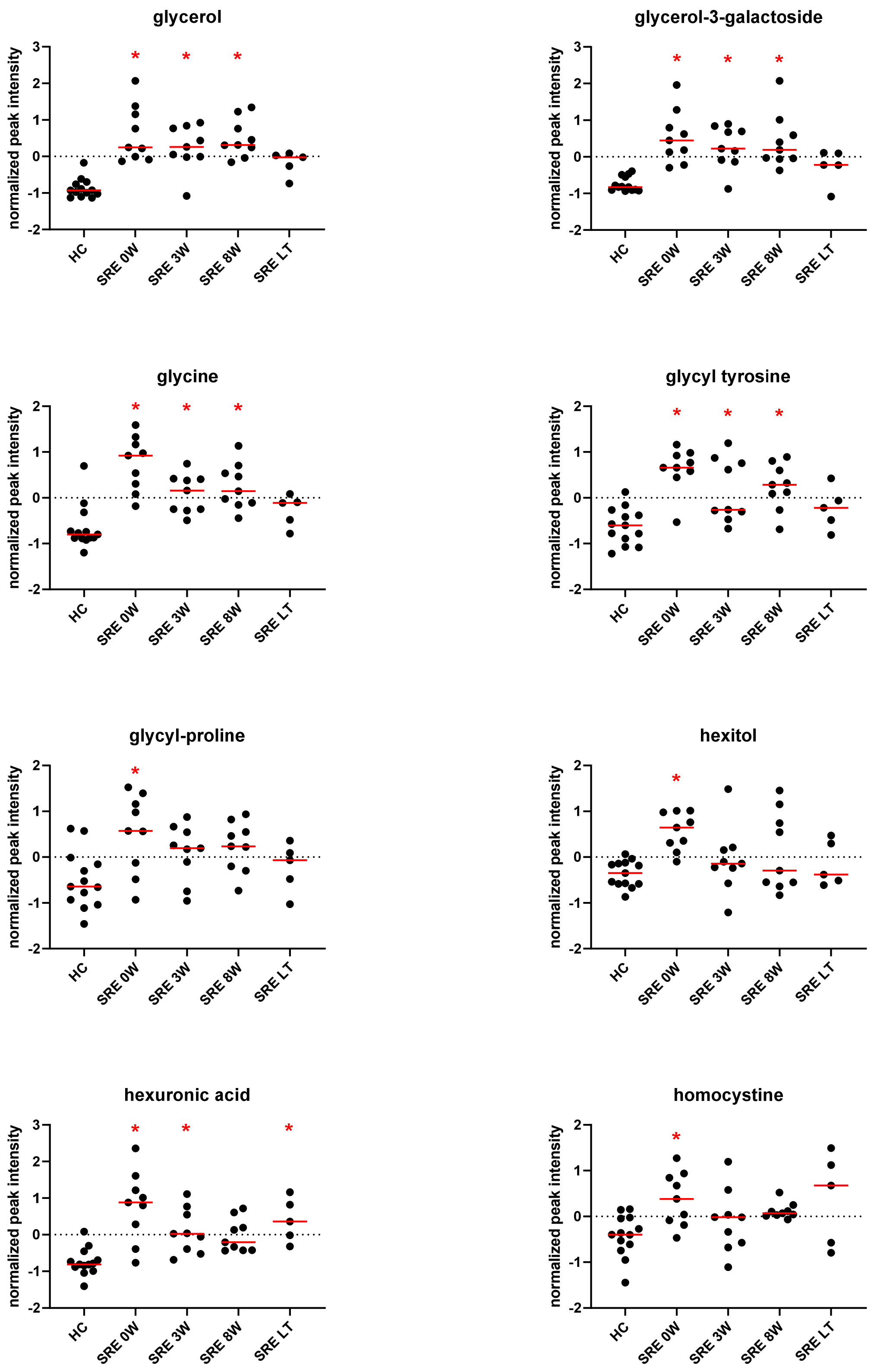
glycerol
glycerol-3-galactoside
glycine
glycyl tyrosine
glycyl-proline
hexitol
hexuronic acid
homocystine
normalized peak intensity
HC
SRE 0W
SRE 3W
SRE 8W
SRE LT
*
3
2
1
0
-1
-2

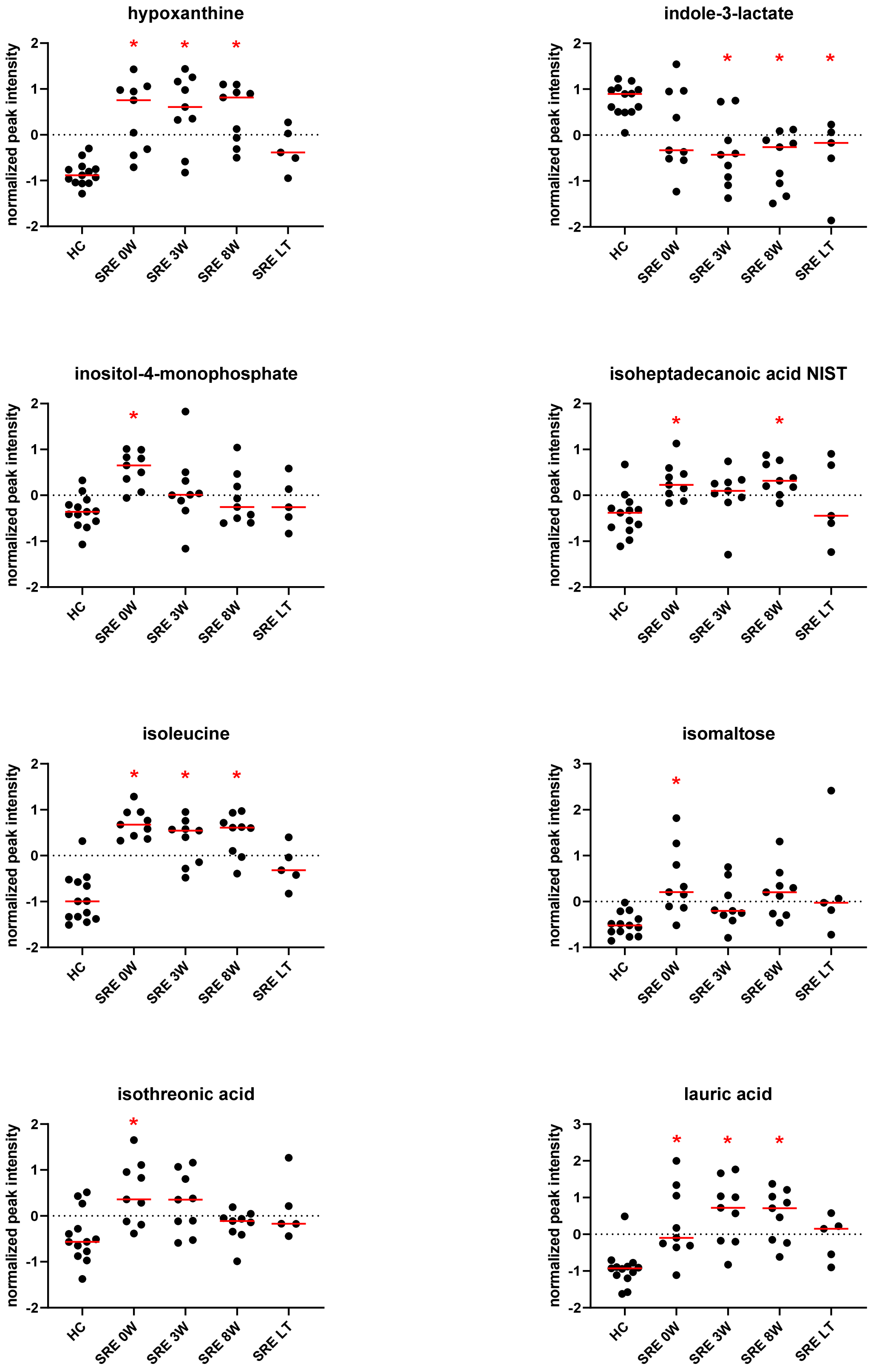
hypoxanthine
indole-3-lactate
inositol-4-monophosphate
isoheptadecanoic acid NIST
isoleucine
isomaltose
isothreonic acid
lauric acid
normalized peak intensity
HC
SRE 0W
SRE 3W
SRE 8W
SRE LT
*

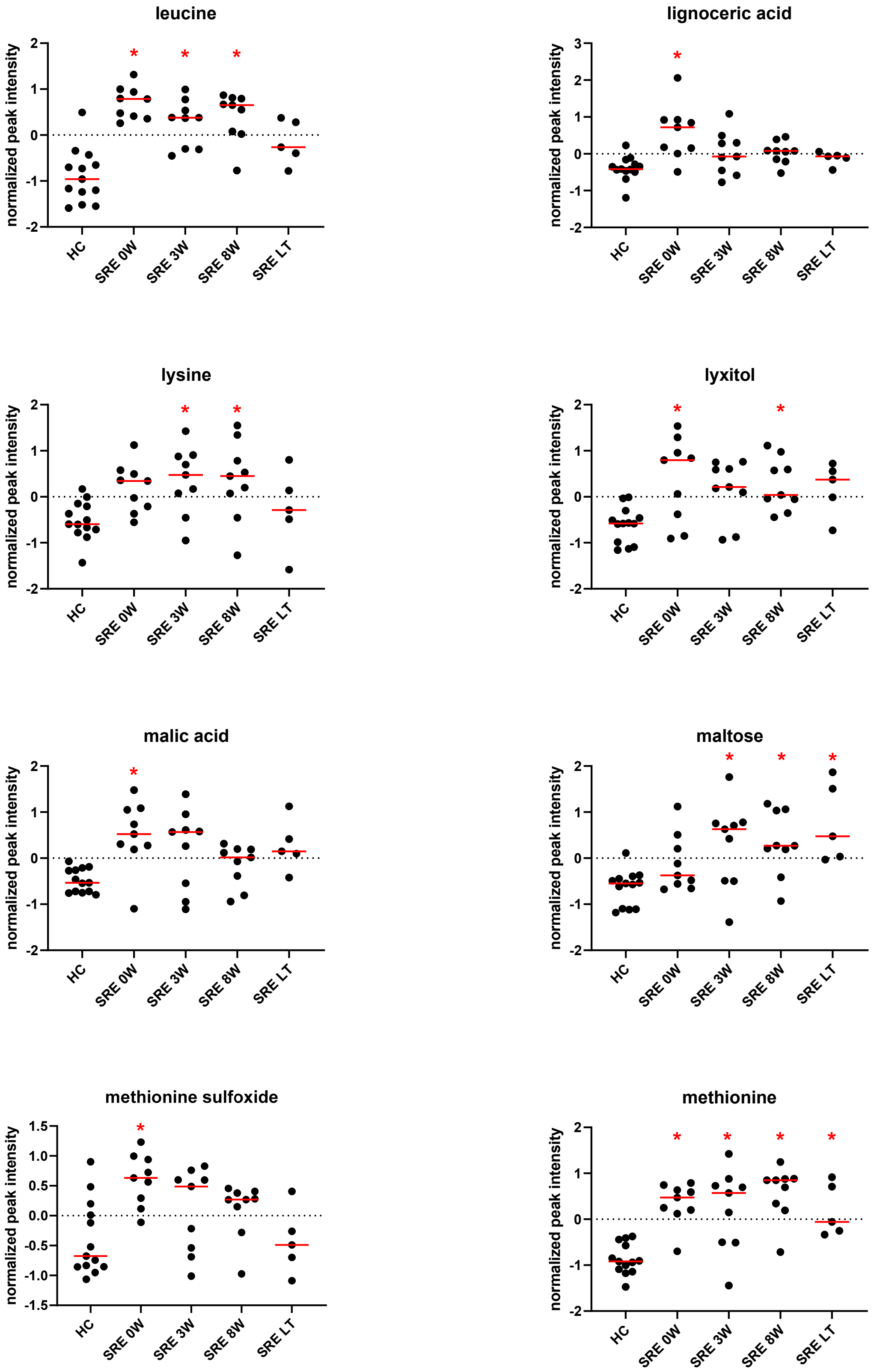
leucine
lignoceric acid
lysine
lyxitol
malic acid
maltose
methionine sulfoxide
methionine
normalized peak intensity
HC
SRE 0W
SRE 3W
SRE 8W
SRE LT
*

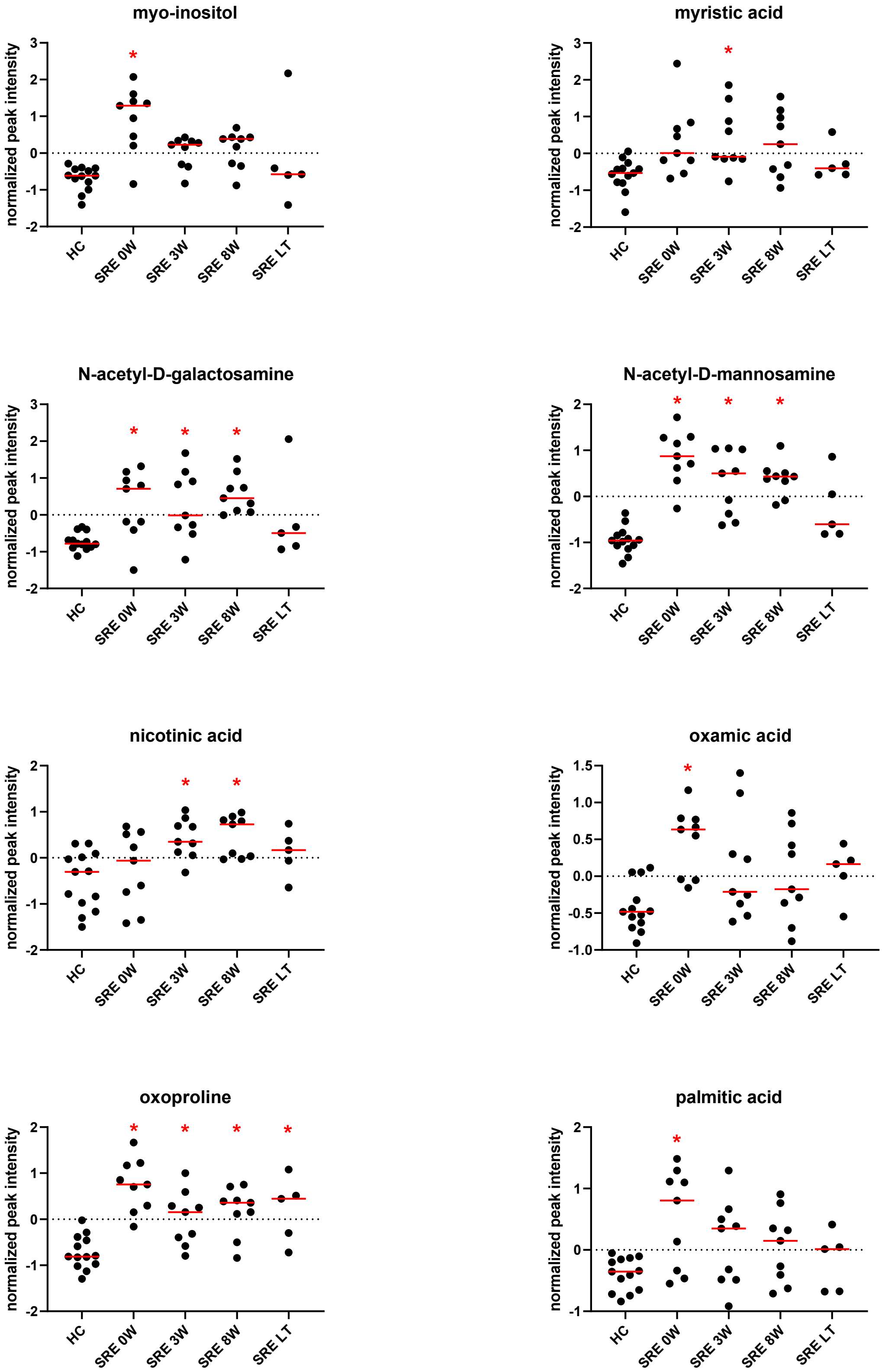
myo-inositol
myristic acid
N-acetyl-D-galactosamine
N-acetyl-D-mannosamine
nicotinic acid
oxamic acid
oxoproline
palmitic acid
normalized peak intensity
HC
SRE 0W
SRE 3W
SRE 8W
SRE LT
*

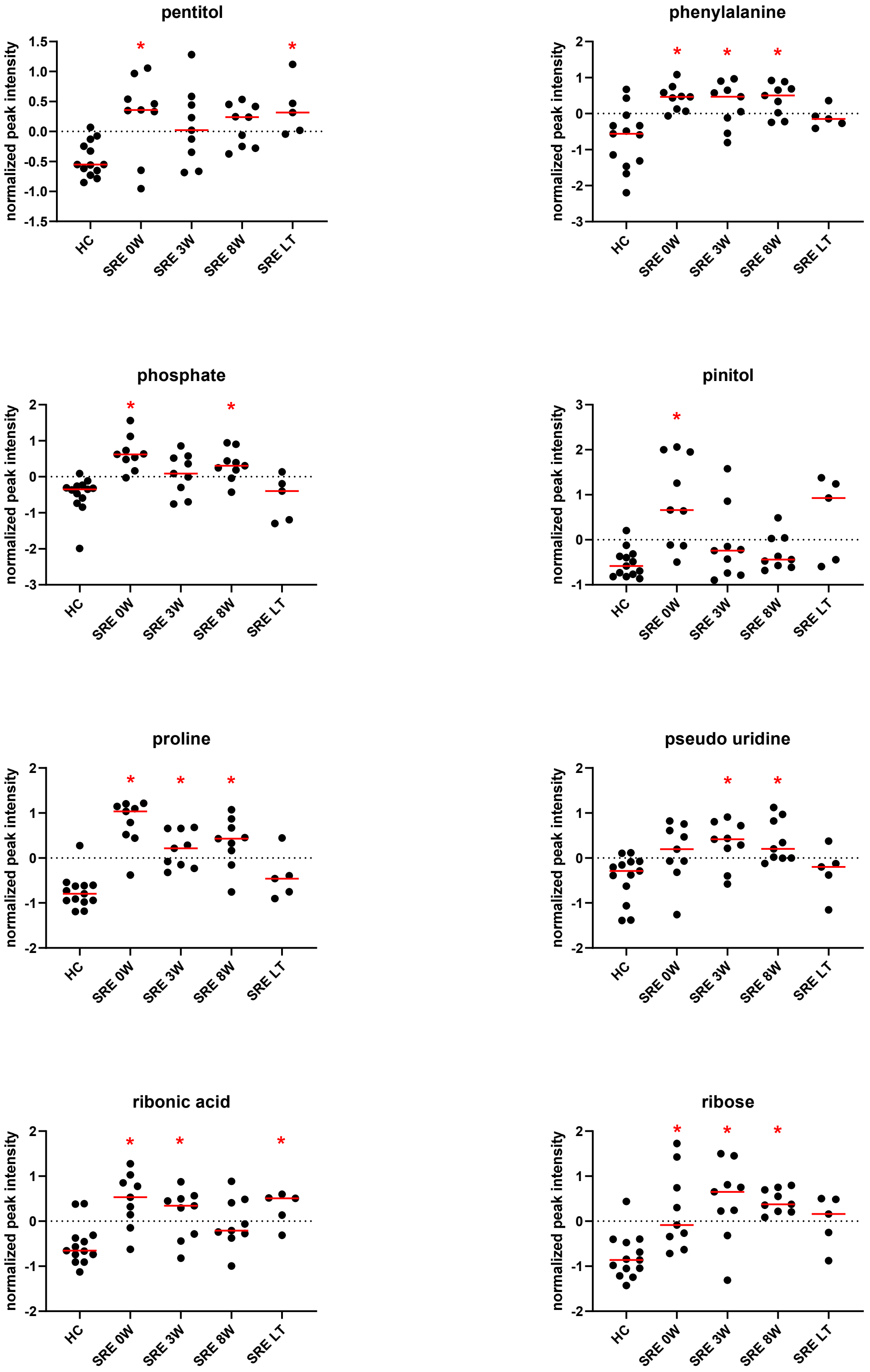
pentitol
phenylalanine
phosphate
pinitol
proline
pseudo uridine
ribonic acid
ribose
normalized peak intensity
HC
SRE 0W
SRE 3W
SRE 8W
SRE LT

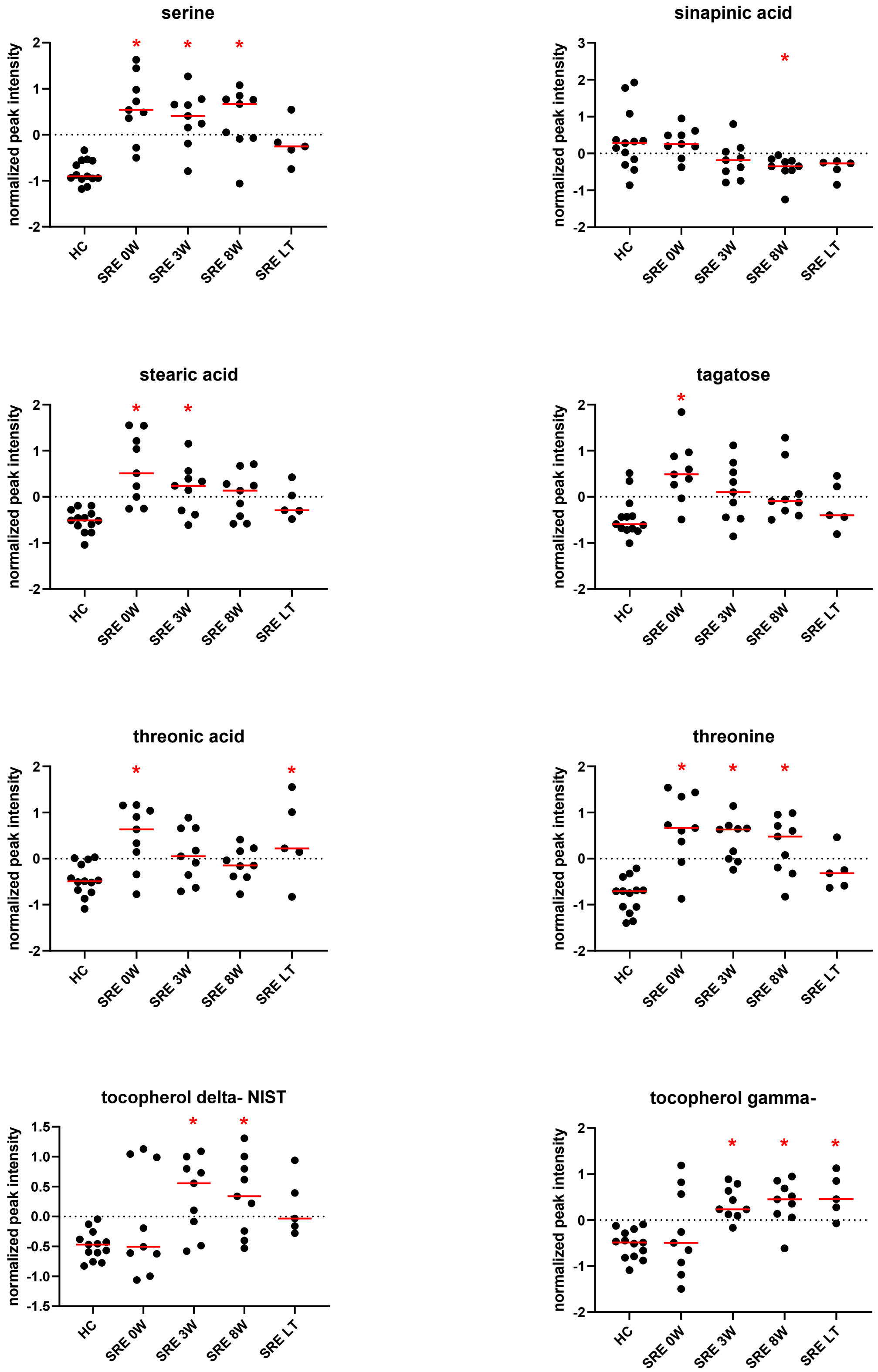
serine
sinapinic acid
stearic acid
tagatose
threonic acid
threonine
tocopherol delta- NIST
tocopherol gamma-
normalized peak intensity
HC
SRE 0W
SRE 3W
SRE 8W
SRE LT
*

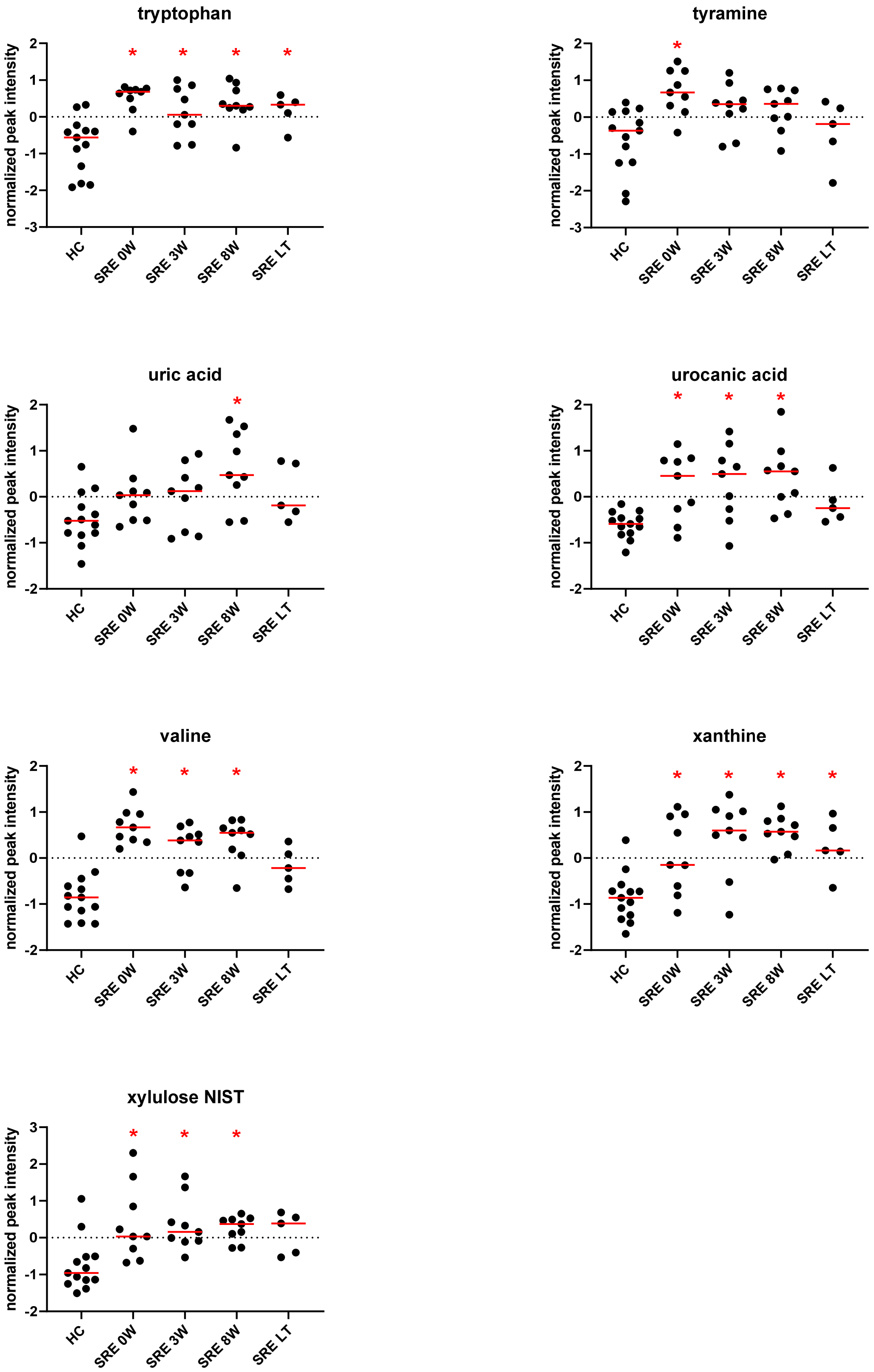

tryptophan
tyramine
uric acid
urocanic acid
valine
xanthine
xylulose NIST
normalized peak intensity
HC
SRE 0W
SRE 3W
SRE 8W
SRE LT
