# Supplementary material for: Long-Term Recovery of the Fecal Microbiome and Metabolome of Dogs with Steroid-Responsive Enteropathy
Source: Animals (Basel). 2021 Aug 25;11(9):2498. doi: 10.3390/ani11092498 (PMC8468387; doi:10.3390/ani11092498)

Supplementary figure S3: Heatmap of the top 25 metabolites found in feces of healthy control dogs, and dogs with SRE over time. Note the less pronounced inter-individual variation in the healthy controls compared to the inter-individual variation in dogs with SRE at baseline.

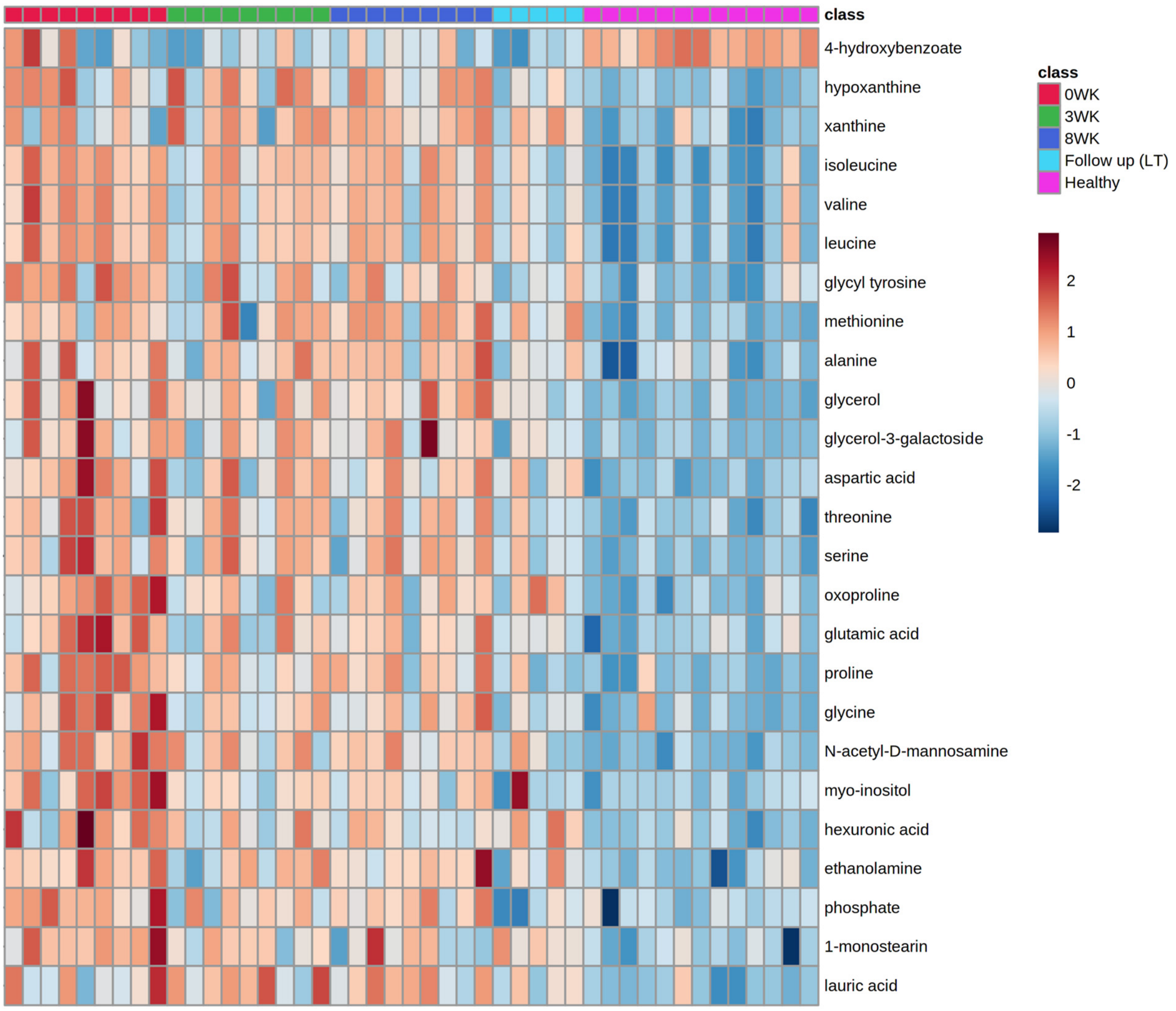

Supplement: Supplementary file 1 [file animals-11-02498-s001.zip › Supplementary_figure_S3_heatmap_individual_samples.pdf]
