# Supplementary material for: Long-Term Recovery of the Fecal Microbiome and Metabolome of Dogs with Steroid-Responsive Enteropathy
Source: Animals (Basel). 2021 Aug 25;11(9):2498. doi: 10.3390/ani11092498 (PMC8468387; doi:10.3390/ani11092498)
Supplement: Supplementary file 1 [file animals-11-02498-s001.zip › Supplementary_table_S3_metabolites.pdf]

Supplementary table S2. Fecal metabolites identified by untargeted metabolomics that were significantly altered by SRE. Compound type, direction of change compared to healthy controls (HC), as well as the time points in which changes were statistically significant are indicated, as well as overall p-values and adjusted p-values (FDR).

Compound name	Compound type	Change in relation to HC	Time points significantly different from HC	p-value	FDR
**alanine**	amino acid	increased	Baseline, 3 weeks, 8 weeks	0.000	0.000
**asparagine**	amino acid	increased	Baseline	0.001	0.006
**aspartic acid**	amino acid	increased	Baseline, 3 weeks, 8 weeks	0.000	0.000
**cysteine**	amino acid	increased	Baseline	0.009	0.029
**cystine**	amino acid	increased	Baseline	0.001	0.003
**glutamic acid**	amino acid	increased	Baseline, 3 weeks, 8 weeks	0.000	0.000
**glycine**	amino acid	increased	Baseline, 3 weeks, 8 weeks	0.000	0.000
**isoleucine**	amino acid	increased	Baseline, 3 weeks, 8 weeks	0.000	0.000
**leucine**	amino acid	increased	Baseline, 3 weeks, 8 weeks	0.000	0.000
**lysine**	amino acid	increased	3 weeks, 8 weeks	0.012	0.037
**methionine**	amino acid	increased	Baseline, 3 weeks, 8 weeks, Long Term	0.000	0.000
**phenylalanine**	amino acid	increased	Baseline, 3 weeks, 8 weeks	0.000	0.002
**proline**	amino acid	increased	Baseline, 3 weeks, 8 weeks	0.000	0.000
**serine**	amino acid	increased	Baseline, 3 weeks, 8 weeks	0.000	0.000
**threonine**	amino acid	increased	Baseline, 3 weeks, 8 weeks	0.000	0.000
**tryptophan**	amino acid	increased	Baseline, 3 weeks, 8 weeks, Long Term	0.000	0.001
**valine**	amino acid	increased	Baseline, 3 weeks, 8 weeks	0.000	0.000
**catechol**	benzenediol	decreased		0.012	0.036
**dehydroabietic acid**	carboxylic acid	increased	Baseline	0.001	0.005

**fucose**	deoxy sugar	increased	Baseline, 3 weeks, 8 weeks	0.003	0.013
**oxoproline**	derivative from amino acid	increased	Baseline, 3 weeks, 8 weeks, Long Term	0.000	0.000
**4-hydroxybenzoate**	derivative of benzoic acid	decreased	Baseline, 3 weeks, 8 weeks, Long Term	0.000	0.000
**malic acid**	dicarboxylic acid	increased	Baseline	0.005	0.018
**oxamic acid**	dicarboxylic acid monoamide	increased	Baseline	0.005	0.019
**glycyl tyrosine**	dipeptide	increased	Baseline, 3 weeks, 8 weeks	0.000	0.000
**glycyl-proline**	dipeptide	increased	Baseline	0.012	0.037
**homocystine**	dipeptide	increased	Baseline	0.018	0.048
**isomaltose**	disaccharide	increased	Baseline	0.010	0.033
**maltose**	disaccharide	increased	3 weeks, 8 weeks, Long Term	0.001	0.006
**3,4-dihydroxyphenyl-acetic acid**	dopamine metabolite	decreased	8 weeks	0.016	0.043
**galacturonic acid**	galactose metabolite	increased	Baseline, 3 weeks, Long Term	0.000	0.001
**glycerol-3-galactoside**	galactose metabolite	increased	Baseline, 3 weeks, 8 weeks	0.000	0.000
**N-acetyl-D-galactosamine**	galactose metabolite	increased	Baseline, 3 weeks, 8 weeks	0.003	0.012
**glucose**	glucose	increased	Baseline, 3 weeks, 8 weeks	0.000	0.002
**6-deoxyglucose**	glucose metabolite	increased	Baseline, 3 weeks, 8 weeks	0.000	0.002
**gluconic acid**	glucose metabolite	increased	Baseline	0.012	0.036
**myo-inositol**	glucose metabolite	increased	Baseline	0.000	0.001
**ribose**	glucose metabolite	increased	Baseline, 3 weeks, 8 weeks	0.000	0.002
**glyceric acid**	glycerol metabolite	increased	Baseline, 3 weeks, 8 weeks	0.000	0.003
**urocanic acid**	histidine metabolite	increased	Baseline, 3 weeks, 8 weeks	0.002	0.010

**2-hydroxyglutaric acid**	hydroxy acid	increased	Baseline	0.014	0.040
**sinapinic acid**	hydroxycinnamic acids	decreased	8 weeks	0.009	0.031
**indole-3-lactate**	indole	decreased	3 weeks, 8 weeks, Long Term	0.000	0.002
**inositol-4-monophosphate**	inositol phosphate	increased	Baseline	0.005	0.019
**2-ketoisocaproic acid**	leucine metabolite	increased	Baseline, 3 weeks	0.000	0.001
**aminomalonate**	metabolism of amino acids	increased	Baseline	0.000	0.002
**2-methylglyceric acid NIST**	metabolite of propionic acid	increased	Baseline	0.006	0.020
**isothreonic acid**	metabolite vitamin C	increased	Baseline	0.005	0.019
**threonic acid**	metabolite vitamin C	increased	Baseline, Long Term	0.003	0.014
**1-monostearin**	monoglyceride	increased	Baseline, 3 weeks, Long Term	0.000	0.001
**fructose**	monosaccharide	increased	Baseline, 8 weeks	0.000	0.003
**N-acetyl-D-mannosamine**	monosaccharide	increased	Baseline, 3 weeks, 8 weeks	0.000	0.000
**tagatose**	monosaccharide	increased	Baseline	0.006	0.022
**xylulose NIST**	monosaccharide	increased	Baseline, 3 weeks, 8 weeks	0.003	0.011
**5,6-dihydrouracil**	nucleic acid metabolite	increased	Baseline, Long Term	0.007	0.022
**adenine**	nucleobase	increased	Baseline	0.016	0.043
**pseudo uridine**	nucleoside isomer	increased	3 weeks, 8 weeks	0.004	0.017
**arachidonic acid**	omega 6 fatty acid	increased	Baseline, 3 weeks, 8 weeks	0.000	0.002
**phosphate**	organophosphate	increased	Baseline, 8 weeks	0.000	0.000
**methionine sulfoxide**	oxidized amino acid	increased	Baseline	0.003	0.013
**3,4-dihydroxyhydro-cinnamic acid NIST**	phytochemical	decreased	Baseline, 8 weeks	0.013	0.037
**glycerol**	polyol	increased	Baseline, 3 weeks, 8 weeks	0.000	0.000

**pinitol**	polyol	increased	Baseline	0.000	0.003
**xanthine**	purine	increased	Baseline, 3 weeks, 8 weeks, Long Term	0.000	0.000
**hypoxanthine**	purine derivative	increased	Baseline, 3 weeks, 8 weeks	0.000	0.000
**uric acid**	purine metabolite	increased	8 weeks	0.013	0.037
**pentitol**	ribose metabolite	increased	Baseline, Long Term	0.004	0.017
**arachidic acid**	saturated fatty acid	increased	Baseline, 3 weeks	0.002	0.009
**caprylic acid**	saturated fatty acid	increased	Baseline, 3 weeks	0.001	0.006
**isoheptadecanoic acid NIST**	saturated fatty acid	increased	Baseline, 8 weeks	0.006	0.022
**lauric acid**	saturated fatty acid	increased	Baseline, 3 weeks, 8 weeks	0.000	0.001
**lignoceric acid**	saturated fatty acid	increased	Baseline	0.001	0.005
**myristic acid**	saturated fatty acid	increased	3 weeks	0.019	0.050
**palmitic acid**	saturated fatty acid	increased	Baseline	0.018	0.048
**stearic acid**	saturated fatty acid	increased	Baseline, 3 weeks	0.000	0.001
**ethanolamine**	serine metabolite	increased	Baseline, 3 weeks, 8 weeks	0.000	0.001
**ribonic acid**	sugar acid	increased	Baseline, 3 weeks, Long Term	0.001	0.006
**hexitol**	sugar alcohol	increased	Baseline	0.013	0.038
**lyxitol**	sugar alcohol	increased	Baseline, 8 weeks	0.004	0.015
**3-(4-hydroxyphenyl) propionic acid**	tyrosine metabolite	decreased	Baseline	0.013	0.037
**tyramine**	tyrosine metabolite	increased	Baseline	0.002	0.009
**3-ureidopropionate**	uracil metabolite	increased	Baseline, 3 weeks, 8 weeks	0.000	0.002
**hexuronic acid**	uronic acid	increased	Baseline, 3 weeks, Long Term	0.000	0.000
**nicotinic acid**	vitamin B3	increased	3 weeks, 8 weeks	0.002	0.008

**tocopherol delta-NIST**	vitamin E	increased	3 weeks, 8 weeks	0.012	0.036
**tocopherol gamma-**	vitamin E	increased	3 weeks, 8 weeks, Long Term	0.000	0.002
**glucoheptulose**		increased	Baseline	0.001	0.006
